# Elementary signaling modes predict the essentiality of signal transduction network components

**DOI:** 10.1186/1752-0509-5-44

**Published:** 2011-03-22

**Authors:** Rui-Sheng Wang, Réka Albert

**Affiliations:** 1 Department of Physics, Pennsylvania State University, University Park, PA 16802, USA; 2 Department of Biology, Pennsylvania State University, University Park, PA 16802, USA

## Abstract

**Background:**

Understanding how signals propagate through signaling pathways and networks is a central goal in systems biology. Quantitative dynamic models help to achieve this understanding, but are difficult to construct and validate because of the scarcity of known mechanistic details and kinetic parameters. Structural and qualitative analysis is emerging as a feasible and useful alternative for interpreting signal transduction.

**Results:**

In this work, we present an integrative computational method for evaluating the essentiality of components in signaling networks. This approach expands an existing signaling network to a richer representation that incorporates the positive or negative nature of interactions and the synergistic behaviors among multiple components. Our method simulates both knockout and constitutive activation of components as node disruptions, and takes into account the possible cascading effects of a node's disruption. We introduce the concept of elementary signaling mode (ESM), as the minimal set of nodes that can perform signal transduction independently. Our method ranks the importance of signaling components by the effects of their perturbation on the ESMs of the network. Validation on several signaling networks describing the immune response of mammals to bacteria, guard cell abscisic acid signaling in plants, and T cell receptor signaling shows that this method can effectively uncover the essentiality of components mediating a signal transduction process and results in strong agreement with the results of Boolean (logical) dynamic models and experimental observations.

**Conclusions:**

This integrative method is an efficient procedure for exploratory analysis of large signaling and regulatory networks where dynamic modeling or experimental tests are impractical. Its results serve as testable predictions, provide insights into signal transduction and regulatory mechanisms and can guide targeted computational or experimental follow-up studies. The source codes for the algorithms developed in this study can be found at http://www.phys.psu.edu/~ralbert/ESM.

## Background

The normal functioning of biological organisms relies on the coordinated action of a multitude of components. The interactions between genes, proteins, metabolites and small molecules form networks that govern gene regulation, determine metabolic rates, and transduce signals [1,2]. Inter-cellular interaction networks such as neuronal networks and immune responses determine organ and organism-level function. High-throughput technologies increase the availability of molecular level data, which enables qualitative and quantitative studies of biological networks [3-6]. At the same time the scarcity of known mechanistic details and kinetic parameters obstructs dynamic (temporal) modeling. There is increasing evidence that the structure of biological interaction networks is closely related to their function [4,7-9]. Therefore, structural and qualitative analysis of biological networks is a promising avenue that takes us closer to a better understanding of their function and evolution [10-15].

Given the topology (i.e. the nodes and edges) of a network, it is natural to wonder just how important (central) each node is to the network's connectivity and functionality. Not surprisingly the issue of node centrality and its correlation with node influence has attracted the attention of many researchers. A large number of graph measures have been developed for evaluating node centrality in complex networks, from degree centrality [16], closeness centrality [17], betweenness centrality [18] to random walk centrality [19], eigenvector centrality [20], spectral centrality measures [21] and communicability measures [22]. A few of these centrality measures have been shown to correlate with the essentiality of genes or gene products in metabolic networks and protein-protein interaction networks [4,23-25].

**Figure 1:**
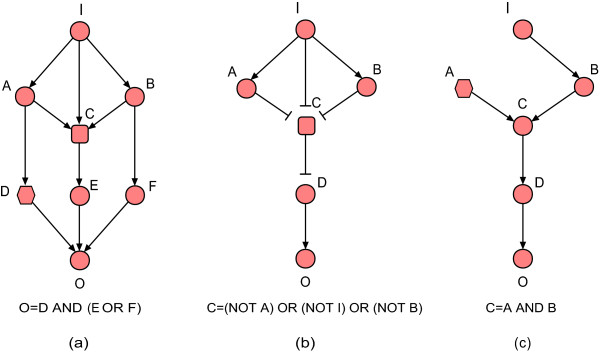
**Illustrative examples for essentiality of components in signaling networks**. I is the input node and O is the output node. All other nodes are intermediate nodes. → denotes activating regulations and ---| represents inhibitory regulations. We use three existing measures of node centrality: betweenness centrality, based on the node's participation in shortest paths between node pairs [18], pairwise disconnectivity index, based on the fraction of initially connected node pairs becoming disconnected after the node's deletion [27], and *SigFlux*, based on the path sets between input and output [29]. (a) E and F are independently activating O, but both of them require the synergy of D to be effective. C (rectangle) has higher betweenness centrality, pairwise disconnectivity index and *SigFlux *value than D. However, D (hexagon) is essential to the signal output, because the disruption of D blocks all signaling paths from I to O. (b) The three regulatory interactions regulating C are conditionally independent. C (rectangle) has very high betweenness centrality, pairwise disconnectivity index and *SigFlux *value. However, knockout of C interrupts its negative action on D, enabling D to activate O. (c) The activation of C requires the presence of both A and B. The *SigFlux *measure is not able to evaluate the importance of components not located on paths from input to output and thus *SigFlux*(A) = 0. However, the regulation of C by A (hexagon) is essential, as the disruption of A blocks the whole signal transduction process.

Typically the functional significance of a gene or gene product is determined by cell viability after its knockout mutation, siRNA interference or inhibition by specific chemical inhibitors. Several recently introduced measures of node importance are based on the effects of the removal of that node on the network's efficiency [17,26] or connectivity [27]. For example, the pairwise disconnectivity index, defined as the fraction of initially connected node pairs which become disconnected after a node and its edges are deleted, was developed to evaluate the importance of gene regulatory network nodes [27]. Yet all currently known graph measures are suited only for describing undirected or directed networks in which the edges denote similar relationships or actions. But to capture the proper biological representation of signaling networks the regulatory interactions denoted by directed edges need also to be distinguished by signs, as they can be either inhibitory or activating interactions. For example, if activation of a transcription factor C requires its freeing from scaffold protein A and its phosphorylation by kinase B, all three nodes have edges toward the activated transcription factor C_p_, of which one, the edge from A to C_p_, is negative (usually denoted as ---|). Moreover, combinatorial regulation is ubiquitous in biological networks; this means that multiple interactions that regulate a component may act in a synergistic (conditionally dependent) fashion [28]. For the above example, since the existence of C_p _requires the presence of B and C and the absence of A, the interactions B → C_p_, C → C_p _and A---|C_p _will be conditionally dependent. This combinatorial nature of regulatory interactions is mostly neglected in graph-based methods developed so far. Even measures specifically designed for signal transduction networks, such as *SigFlux *[29], ignore such negative regulation and conditional dependency. These methods, based on path analysis, may take into account structural redundancy that in fact is not functional due to conditional dependency. Furthermore, they cannot resolve ambiguous dependencies, namely inconsistent paths caused by inhibitory regulations [10]. Finally, in existing structure-based methods, gene knockouts are simulated by simply deleting the corresponding node and the edges incident on it [12,26,27]. However, due to conditional interdependence a node may be required for the functioning of other downstream nodes, therefore the disruption of any single node may lead to a cascading breakdown of a large part of the system. Ignoring inhibitory interactions, synergistic regulations and cascading effects will lead to biased results (see Figure 1).

In this work, we develop a novel method that addresses the shortcomings listed above. Our method expands a signaling network to a new representation that incorporates the sign of the interactions as well as the combinatorial nature of multiple converging interactions. We then simulate both knockout and constitutive activation of components as node disruptions, and determine the possible cascading effects of a node disruption by identifying indispensable regulatory effects. We introduce the new concept of elementary signaling mode (ESM), as being the minimal set of nodes that can perform signal transduction independently. The importance of each signaling component is then determined by comparing the number of ESMs following the cascading disruptions caused by the removal of the component with the number of ESMs in the intact network. We apply this method to several signaling networks including a network describing the immune response of a mammalian host to bacteria [30], a guard cell abscisic acid (ABA) signaling network in plants [31], and a T cell receptor signaling network [32]. The results demonstrate that our method can effectively uncover the essential signaling components mediating a signal transduction process. The essentiality of signaling components predicted by our method is in strong agreement with the results of Boolean (logical) dynamic models and experimental observations. Our approach incorporates both inhibitory and synergistic interactions in structural analysis and can be used effectively to other types of regulatory networks.

## Results

### Integrative evaluation of the essentiality of signaling components

Signaling networks can be represented as directed graphs in which nodes denote signaling components, edges represent regulatory interactions, and the orientation of the edges reflects the direction of signal transduction [10,29-31,33]. The input (source) nodes of signaling networks represent the initial signals or their receptors, the intermediate nodes consist of various kinases and second messengers, and the output (sink) nodes represent transcription factors or cellular responses. The edges of signaling networks generally represent directional interactions such as phosphorylation, transcriptional regulation, and enzyme catalysis, which result in either inhibitory or activating effects. Our general aim is to predict the essentiality of signaling components through structural (graph theoretical) analysis. Since the graph corresponding to a signaling network does not reflect the possible conditional dependency between incoming edges and cannot resolve inconsistent paths caused by inhibition, we propose an augmented graph representation that naturally incorporates synergy and inhibition. Our method is based on three main steps: network expansion, determination of the cascading effects of node removal, and using the novel concept of elementary signaling mode (ESM) to characterize the network before and after removing a node. The graph theoretical framework proposed here is uniquely suited to signaling networks and similar regulatory networks in which the edges do not necessarily correspond to elementary reactions.

#### Expansion of signaling networks

**Figure 2:**
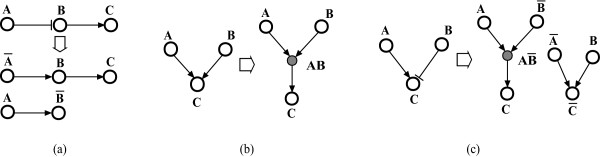
**Operation rules for the expansion of signaling networks **(a) A inhibits B, so two complementary nodes  and  are introduced as the logical negations of A and B, respectively. The inhibitory edge from A to B is replaced with an activating edge from A to  and an activating edge from  to B. (b) The interactions from A and B to C are conditional on each other, so a composite node AB is introduced. The activation of AB depends on the presence of A and B. The activation of C depends on AB. (c) The activation of C requires both the presence of A and the absence of B. The two operations illustrated in (a) and (b) are executed in combination to expand a signaling network.

We utilize two operation rules to expand a signaling network to a new representation that incorporates the regulatory logic (e.g. inhibition, synergistic regulations). First, to take into account inhibitory interactions, we introduce a complementary node for each component that negatively regulates other nodes or is being negatively regulated by other nodes (see Figure 2(a)). This complementary node represents the logical negation of the original node, and allows us to evaluate the influence of the original node's inhibitory effect on the output node. Since nodes which are activated by others and have only activating effects on other nodes have no direct inhibitory roles, we do not introduce complementary nodes for them. An inhibitory edge starting at a node in the original network becomes an activating edge starting at its complementary node in the expanded network. Similarly, an inhibitory edge ending at a node becomes an activating edge ending at its complementary node. Introducing complementary nodes may lead to edges or subgraphs with no connections or relevance to the paths between input(s) and output(s). These edges or subgraphs are discarded by traversing the expanded network from input(s) to output(s) and keeping only the nodes and edges that are relevant to at least one input-output path.

Second, we introduce composite nodes to incorporate conditionally dependent relationships. We represent the combinatorial relationship of multiple regulatory interactions converging on a node *v *by a Boolean rule (see Methods) which can be uniquely written in the following disjunctive normal form:

where *u_ij _*are regulators of node *v*. Usually the Boolean rules for each node will need to be constructed individually on the basis of the existing biological evidence. Our method can be used even if only partial information of inhibitory regulations and synergistic interactions is available (see Methods for guidelines). The Boolean rule governing a complementary node is the logical negation of the Boolean rule that governs its corresponding original node. For each set of synergistic interactions (with AND relationship) ending at a node, we introduce a composite node to denote the synergy in a graphical form [34,35]. The regulators of *v *activate the composite node, which then activates the node *v *(see Figure 2(b)). The two operations illustrated in Figure 2(a) and 2(b) are executed in combination to expand the signaling network (see Figure 2(c)).

Introducing complementary nodes and composite nodes increases the number of nodes and edges in the network, but the benefit is that ambiguity is eliminated. All the directed interactions in the expanded network represent activation. Multiple edges ending at a composite node are conditionally dependent on each other, and multiple edges ending at an original node or complementary node are independent. The expanded signaling network does not have ambiguous dependencies, and can serve as a substrate for augmented structural methods. In addition, expansion of a signaling network by decomposing complex Boolean rules into independent elements helps to untangle the network into individual modules.

#### Cascading effects of a signaling component's removal

**Figure 3:**
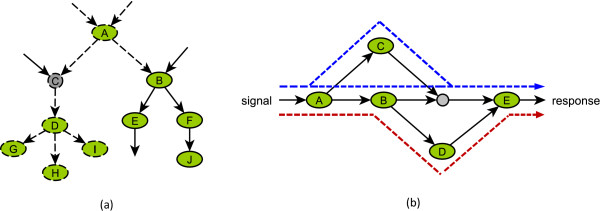
**Illustration of the cascading effects of a component's deletion and of the elementary signaling modes in signaling networks.** (a) The cascading effects of a component's deletion. (b)  Elementary signaling modes. In (a) and (b), the ovals denote original nodes or complementary nodes, and the small circles mean composite nodes. In (a), the dashed edges and the dashed node contours indicate the nodes and edges that will be disrupted in the cascading failure following the removal of node A. In (b), the dashed shapes outline two elementary signaling modes (ESMs). Note that different ESMs can have common nodes.

As the expanded signaling network clearly represents the relationships among nodes and signaling interactions, we can evaluate the essentiality of a signaling component by examining the range to which its perturbation propagates. We determine the cascading effect of the removal of a node by an algorithm that iteratively finds and deletes the nodes that have just lost their indispensable regulators (see Methods). There are three cases for a regulator *v *to be indispensable for a direct target node *u*: (1) *v *is the sole regulator of *u*; (2) *u *is a composite node; (3) *v *is the only remaining regulator of *u *left due to the disruption of other regulators. Figure 3(a) shows an example in which removal of node A leads to the disruption of C, D, G, H, and I, but does not eliminate nodes B, E, F and J, since there are two independent signaling interactions activating B. Removing an original node simulates the knockout of a signaling component and evaluates the importance of the activating role of this component. In contrast, removal of a complementary node simulates the constitutive expression (activation) of the signaling component and evaluates the influence of the inhibitory role of this component.

#### Elementary signaling modes

The connectivity of a signaling network between the input node(s) (e.g. ligands) and the output node(s) (e.g. cellular responses) is most reflective of its signal transduction capacity. The concept of shortest paths is widely used to characterize the efficiency of information flow or communicability in networks [17]. However, this measure would classify as unimportant all the nodes that are located outside of such shortest paths, which is clearly unrealistic for signaling networks.

Elementary flux modes, minimal sets of enzymes that can make a metabolic system operate at a steady state, play an important role in metabolic network analysis [8,36]. Previous efforts for adapting this concept to signaling networks apply to networks that include only chemical reactions and activating regulation [37] or to enzyme cascades (e.g. phosphorylation and dephosphorylation) [38]. Here we propose a counterpart of elementary flux modes in signaling networks which include a variety of interactions and regulatory relationships. An elementary signaling mode (ESM) is defined as a minimal set of components that can perform signal transduction from initial signals to cellular responses. By 'minimal', we mean that an ESM is not decomposable and none of its signaling components is redundant, i.e. knockout of any of the nodes in the ESM will make it unable to transduce the signal. The concept of ESM is an extension of the graph concept of simple path. An ESM that does not contain any composite nodes is indeed a simple path. If the ESM has a composite node, it additionally includes all the edges ending at the composite node and their upstream nodes (see Figure 3(b)). We formulate the identification of an ESM into an integer linear programming problem and design an iterative algorithm to calculate all ESMs in a signaling network (Additional file 1). Since integer programming is NP-hard and cannot be used to enumerate all ESMs in large networks, we also design an efficient depth-first search-based approximate algorithm for estimating the number of ESMs in a signaling network (Additional file 1). In addition, we determine the shortest ESM as an extension of the concept of shortest path by using a dynamic programming procedure (see Methods).

Our method ranks the importance of signaling components by the effects of their perturbation on the ESMs of the network. We characterize each node *v *by the relative reduction in the number of ESMs following the cascading loss of nodes caused by the removal of *v *(Methods). This measure takes values in the interval [0,1], with 1 indicating a node whose loss causes the disruption of all ESMs between the input and output node(s). As a comparison we also define the analogous measure using simple paths instead of ESMs (Methods).

### Validation on three signaling networks

Several regulatory networks with documented synergistic and inhibitory interactions have been published [30-33,35,39], which are suitable for validation of our method. We choose three signaling networks describing the immune response of mammals to bacteria, guard cell abscisic acid signaling in plants, and T cell receptor signaling [30-32], as benchmarks for validating our method. We use the Boolean rules developed in prior articles on these networks to encode synergistic interactions and inhibitory regulations. The essentiality of each real node and complementary node is determined by using our ESM centrality measure (denoted by *E*^ESM^) and the simple path (SP) centrality measure (denoted by *E*^SP^). We compare these measures to a traditional centrality measure, node betweenness centrality (denoted by BC) [18], as well as the simple path measure used without considering the cascading effects of a node deletion (which is equivalent to the *SigFlux *measure [29]). We evaluate the importance values given by each method by comparing with experimental observations. Additionally we characterize each method's classification accuracy by comparing with the results of Boolean (logical) dynamic models (Additional file 2). Specifically, components are classified as essential if their knockout (OFF state) or constitutive activation (ON state) leads to an incorrect state of the output (Additional file 2). The effect of deleting an original node (a complementary node) in our method is compared with that of keeping this node as OFF (ON) in the Boolean dynamic models or logical steady state analysis. We use sensitivity (the fraction of essential components that are recognized by a method) and specificity (the fraction of non-essential components that are recognized by a method) to evaluate the four methods. High sensitivity with high specificity (i.e. low false discovery rate) indicates good performance of a method. Varying the threshold of importance values that corresponds to essentiality gives a series of sensitivity and specificity values that form an ROC curve.

#### The host immune response network

Thakar *et al. *assembled a regulatory network of immunological processes activated upon invasion by *Bordetellae *bacteria and developed asynchronous Boolean dynamic models of bacterial infections [30]. This network has 18 nodes, of which 'bacteria' can be considered as the input node, and 'phagocytosis' as the output node. The sixteen intermediate nodes include innate immune components such as pro-inflammatory cytokines and dendritic cells, early induced immune components such as B cells, and adaptive immune components such as T helper cells and related cytokines.

**Figure 4:**
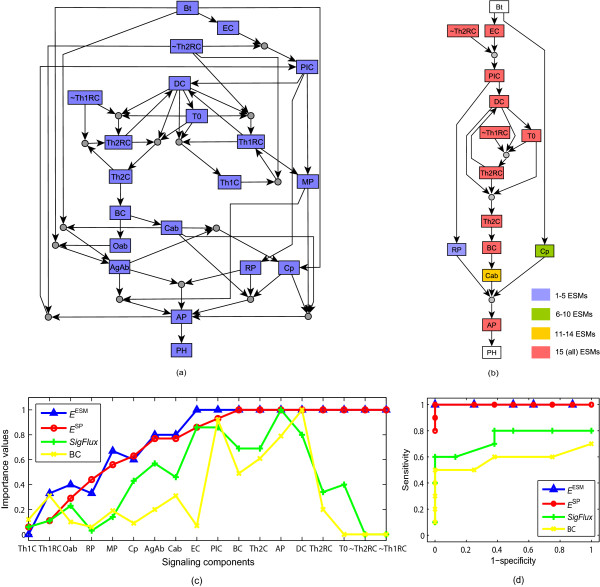
**Results on the immune response network. **(a) The expanded host immune response network. (b) The shortest elementary signaling mode in this network. (c) Importance values of nodes in this network obtained by single-node deletions. (d) Prediction accuracy. Bt: bacteria, EC: Epithelial cells, PIC: Pro-inflammatory cytokines, Th1RC: T helper cell type 1 related cytokines, Th2RC: T helper cell type 2 related cytokines, DC: Dendritic cells, T0: T0 cells, Th1C: T helper cell type 1, Th2C: T helper cell type 2, MP: Macrophages, BC: Antibody-producing B cells, Cab: Complement-fixing antibodies, Oab: Other antibodies, AgAb: Antigen-antibody complex, RP: Recruited PMNs, Cp: Complement, AP: Activated phagocytes, PH: Phagocytosis. In (a) and (b), composite nodes are represented by small gray solid circles, original nodes and complementary nodes are represented by rectangles. The labels of complementary nodes are denoted by the labels for the corresponding original nodes prefixed by the symbol '~'. The color coding of the nodes in (b) indicates the level of their participation in the 15 ESMs of the network. In (c) and (d), triangles represent the importance values or prediction accuracy obtained by the ESM measure, circles represent the simple path measure, plus signs denote the *SigFlux *measure, and crosses show the betweenness centrality measure.

Using this network and its Boolean rules, we construct the expanded host immune response network shown in Figure 4(a). Three time-dependent details of the Boolean rules, namely timed decay for Th1RC and Th2RC, the threshold effect in the Boolean rule for T0, and the self-regulations in the Boolean rules for Cab and Oab are not included in the expanded network. Although the Thakar et al. network includes a negative edge between phagocytosis (PH) and bacteria (Bt), which can be translated into the edge ~PH→Bt, this edge is not relevant to the process from the input Bt to the output PH and thus is not included in the expanded network. Due to the relatively small size of the host-immune response network we can exactly enumerate all ESMs. The integer linear programming algorithm and the depth-first-search algorithm using the multiplication operation (see Additional file 1) agree in indicating that there are 15 ESMs in this network. We can see from Figure 4(b) showing the shortest ESM that half of the nodes are involved in all ESMs and are therefore essential to bacterial clearance. The flexible signaling components including Cab, AgAb, Cp, MP, RP, Oab, Th1RC, are involved in a varying number of ESMs.

The importance values of signaling components based on the ESM measure and the SP measure are shown in Figure 4(c). Both measures indicate that single-node deletion of six components, BC, Th2C, Th2RC, T0, DC or AP, leads to the elimination of all signal transduction from bacterial infection to bacterial clearance. These predictions are validated by the experimental observations [40-42] indicating that the deletion of B cells (BC), T0 cells (T0), dendritic cells (DC) or a lack of adaptive immune response results in bacterial persistence. The main difference between the results of the ESM measure and the SP measure lies in the importance values of EC and PIC. Knocking out EC or PIC disrupts all ESMs, but there are several simple paths left. The essentiality of these two nodes indicated by the ESM measure is supported by the fact that pro-inflammatory cytokines and inflammation are required for the resolution of infections [43]. Although betweenness centrality and *SigFlux *give importance values that correlate with those obtained by our method for some components, they fail to capture the essentiality of several other components such as Th2RC and T0 cells. In addition, single-node deletion of the complementary nodes of Th1RC or Th2RC will completely block bacterial clearance according to both the ESM and SP measure, indicating that the inhibition of these nodes at certain stages of the infection is essential for the immune response. Indeed, experimental observations confirm that a switch-over between Th2-related and Th1-related immune functions is necessary for bacterial clearance [30,40]. However, betweenness centrality and the *SigFlux *measure give low importance values for these inhibitory nodes, which contradict immunological knowledge.

We rerun the Boolean dynamic model of Thakar *et al. *[30] to perturb each component and obtain its essentiality (Additional file 2); the results are given in Table S1 in Additional file 2. The prediction accuracy as compared to the dynamic model obtained by the four graph measures is shown in Figure 4(d). One can clearly see that the ESM measure and the SP measure which incorporate information from inhibitory regulation and synergistic relationships can fully capture the essentiality of signaling components and have a much better performance than betweenness centrality and *SigFlux*.

#### The guard cell ABA signaling network

Plants take up carbon dioxide for photosynthesis and lose water by transpiration through pores called stomata, which are flanked by pairs of specialized guard cells. The size of stomata is regulated by the guard cells' turgor [44]. Under drought stress conditions, plants reduce water loss by synthesizing the phytohormone abscisic acid (ABA) that triggers stomatal closure. Li *et al. *[31] assembled a signaling network corresponding to ABA-induced stomatal closure and formulated an asynchronous Boolean model of the process. There are over 50 nodes in this network, with one input, ABA, and one output, stomatal closure. The intermediate nodes include signaling proteins such as the G protein α subunit GPA1, second messengers such as cytosolic Ca^2+^, phosphatidic acid, as well as ion channels.

**Figure 5:**
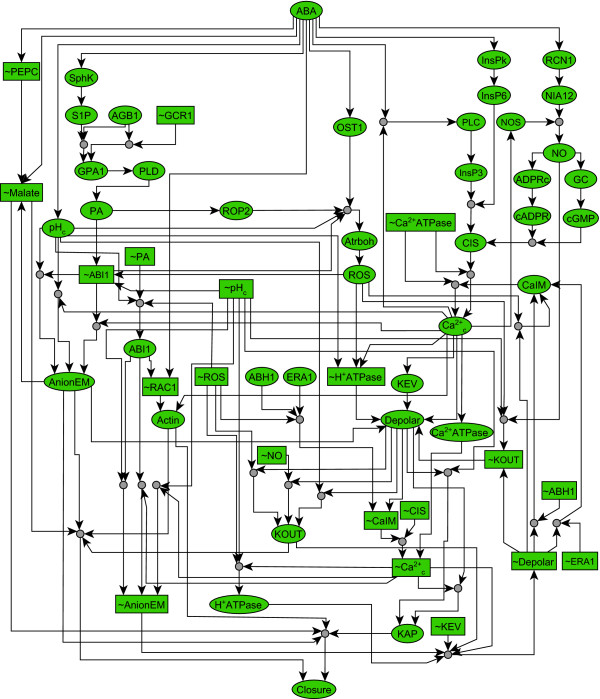
**The expanded guard cell ABA signaling network**. Composite nodes are represented by small gray solid circles, original nodes are represented by large empty ovals, and complementary nodes are represented by rectangles. The labels of complementary nodes are denoted by the labels for the corresponding original nodes prefixed by the symbol '~'.

Using this network and the Boolean rules, we construct the expanded ABA signaling network shown in Figure 5. The shortest ESM from ABA to Closure has 21 nodes and has a length of 11. The importance values of signaling components arising from single-node deletions are summarized in Figure S1, where the number of ESMs is calculated by the depth-first-search algorithm using the max operation (Additional file 1). Our method shows that knockout of AnionEM, Depolar or Actin will completely block all the signaling paths and ESMs. Disruption of other components such as GPA1, AGB1, CaIM, PLD, PA, pH_c_, H^+^ATPase, Ca^2+^_c_, or KOUT leads to a strong reduction of the signal transduction connectivity. In addition, single-node knockouts of SphK and S1P will increase the length of the shortest ESM by more than 60%, suggesting that these signaling components are critical for the efficiency of ABA signal transduction. The important components predicted by our method are validated by numerous experimental observations (Additional file 3).

**Figure 6:**
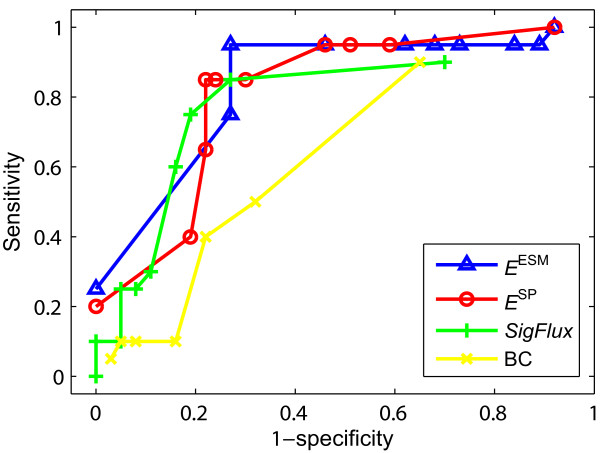
**Comparison of different methods applied to the guard cell ABA signaling network in terms of prediction accuracy**. Triangles represent the prediction accuracy of the ESM measure, circles represent the simple path measure, plus signs denote the *SigFlux *measure, and crosses show the betweenness centrality measure.

A detailed comparison of prediction accuracy by the four methods is given in Figure 6, where we use the perturbation results of the Boolean dynamic model as the standard (Additional file 2, Table S2). By comparing with the dynamic simulation result, the best accuracy of the ESM measure is 95% sensitivity, 73% specificity (for an importance threshold of 0.8). The best performance of the SP measure is a sensitivity of 85% and specificity of 78% (for a threshold of 0.6). The best accuracy of the *SigFlux *measure is sensitivity 85%, specificity 73% while that of the betweenness centrality is sensitivity 50%, specificity 68% (both for a threshold of 0.1). Again, the performances of the ESM measure and the SP measure are better than those of the other two. We also evaluate the importance of two-node combinations by simultaneously deleting two original nodes, two complementary nodes, or an original node and a complementary node. The results, shown in Figure S2, are highly consistent with the dynamic simulation in Li *et al. *2006 [31] (Additional file 3).

#### The T cell receptor signaling network

T cells (lymphocytes) play a central role in the immune response. T cells detect antigens by a special receptor on their surface called T cell receptor (TCR), which is triggered by Major Histocompatibility Complex (MHC) molecules and induces a series of intracellular signaling cascades. CD28 provides an essential co-stimulatory signal during T-cell activation, which increases T cell proliferation and prevents the induction of anergy and cell death. Saez-Rodriguez *et al. *constructed a 94-node T cell receptor signaling network with three input nodes and seven output nodes [32] and used the software CellNetAnalyzer [11] to calculate the logical steady states of this network.

We use CD28 antigen and the ligand of the T cell receptor (denoted by TCRlig) as the two inputs of the T cell signaling network and use NFκB and AP1 as the two outputs. The other outputs studied by Saez-Rodriguez *et al. *are implicitly incorporated in this analysis as the connectivity from the inputs to SRE, CRE and p38 is contained in that from the inputs to AP1, and the connectivity from the inputs to NFAT and PKB is contained in that from the inputs to NFκB. We use our method to expand this T cell receptor signaling network into a new representation shown in Figure S3 (Additional file 4).

**Figure 7:**
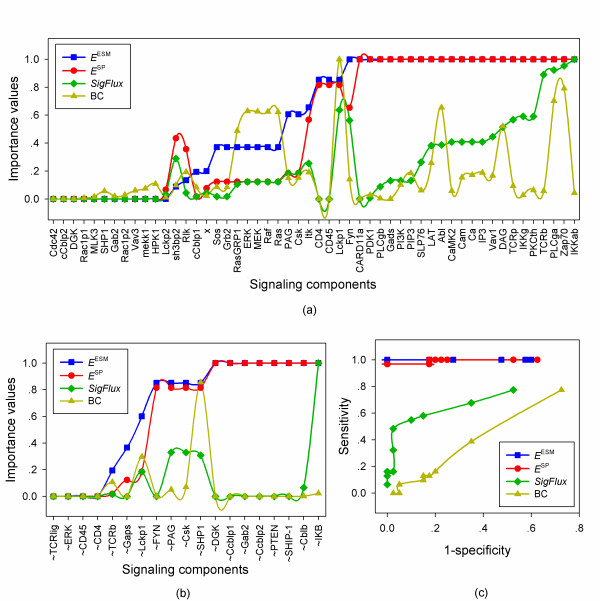
**Comparison of different methods applied to the T cell receptor signaling network with NFκB as the output**. (a) Importance values obtained by single-node deletion of original nodes. (b) Importance values obtained by single-node deletion of complementary nodes. (c) Prediction accuracy. Rectangles indicate the importance values or prediction accuracy obtained by the ESM measure, circles represent the simple path measure, diamonds denote the *SigFlux *measure, and triangles show the betweenness centrality measure.

The importance values of signaling components obtained by single-node deletions are summarized in Figure 7(a) and Figure 7(b), respectively, where the number of ESMs is calculated by the depth-first-search algorithm using the max operation (Additional file 1). Our method shows that more than 20 components are essential to the activation of the transcription factor NFκB, as single-node disruption of these components blocks all the signaling paths and ESMs to NFκB. Most of these components are located in the core of the T cell receptor signaling network [32]. The importance values given by the ESM measure and the SP measure are very similar except the difference in evaluating the node Fyn, whose ESM-given essentiality is supported by the logical steady state analysis. In contrast, *SigFlux *and betweenness centrality cannot recognize the core part of the T cell signaling network.

We calculate the steady states of this T cell receptor signaling network [32] by using the software CellNetAnalyzer [11] (Additional file 2). The essentiality of signaling components obtained from the perturbation results is summarized in Table S3 (Additional file 2). We can see from Figure 7(c) that both the ESM measure and the SP measure capture well the essentiality of the T cell receptor signaling components, whereas the other two methods do not. The results by using AP1 as the output, given in Figure S4 (Additional file 4), also support this conclusion.

## Discussion

In this study, we propose a method for quantifying the importance of components in signaling and regulatory networks. This method incorporates synergistic and inhibitory regulation that is quite common in signaling networks but has received little attention so far in structural analysis. Our method can be easily adapted for evaluating the importance of genes in gene regulatory networks by considering the connectivity of the whole network instead of the connectivity from input to output. In addition, our graph measures can be readily adapted to evaluate the importance of edges (interactions). This allows the study of mutations of binding sites that do not knock components out but change their interactions [45].

While ESMs are the most concise and complete description of the signal transduction modes in a network, the combinatorial aspects of ESMs also make them difficult to count in large networks. Our results indicate that the simple path (SP) measure has a similar performance as the ESM measure as an indicator of node centrality. The reason is that both ESM and SP measures incorporate the cascading effects of a node's removal arising from the synergistic relations between multiple interactions. Either measure can serve the purpose of identifying a few most important components in a signaling network. The integer linear programming algorithm proposed in this study can be used by those researchers interested in individual signaling modes.

In addition to the application described in this study, ESMs can also be used to probe the relationship between the structure and dynamics of a signaling network. For example, if the dynamics of a signaling network is oscillatory, the state of at least one node needs to switch from 0 to 1 and vice versa, and thus it is possible that some ESMs contain both an original node and its complementary node. Thus one may predict the potential dynamics of the signaling network from the composition of its ESMs. The minimal intervention set, defined as a minimal set of important nodes whose simultaneous manipulation satisfies a user-defined goal (e.g. permanent deactivation of the output) [10,46], identifies minimum failure modes for signaling networks and regulatory networks. One can conjecture that any node or minimal node combination whose deletion disrupts all the ESMs may be a minimal intervention set. For the example in Figure 3(b) a sustained signal (i.e. stable ON state of the input node) leads to a sustained response according to logical steady-state analysis [10]. There are three minimum intervention sets of size 1: {A}, {B}, and {E}, whose knockout (maintained OFF state) blocks the signal transduction and eliminates the response. Single-node deletion of A, B, or E disrupts all ESMs in this example, supporting the conjecture. Unlike the minimal failure modes defined by minimal intervention sets, the ESM measure gives quantitative importance values for all signaling components, regardless of whether they are important or not. The detailed relation between the dynamics of a signaling network and its ESMs is an interesting topic worth exploring in future research.

Our method requires less prior information such as initial conditions and timing, has less computational cost and performs as well as methods involving dynamic simulations such as Soni *et al. *2008 [47]. Soni *et al. *constructed an ensemble of Boolean network simulations to estimate the frequency of active pathways and to rank interactions by their control effective flux (CEF). Since the same guard cell ABA signaling network was used as test example in their study, we can compare their results with ours. There are 7 intermediate signaling components involved in the five interactions with the highest CEF values. 5 of them have very high importance values (>0.98) according to our ESM measure, 4 of which are essential components according to dynamic simulation. In contrast, the remaining 2 signaling components have low importance values (<0.5) according to our ESM measure, and the dynamic simulation also shows that their knockout does not affect ABA signal transduction.

Another related work by Abdi *et al. *applies digital circuit fault diagnosis methods to generic Boolean representations of signaling networks to find vulnerable signaling components [48]. The method determines the probability that an error occurring at a signaling component propagates to the output(s) by calculating the signal probability (the probability of the state 1) of all nodes on the paths from the error site to the output(s). Vulnerable components are those nodes that have high error propagation probabilities to the output(s). A comparison on two signaling networks they used (the caspase3-FKHR network and the p53 network) indicates that signaling components identified as vulnerable (error propagation probability > 0.5) by Abdi *et al. *tend to have high essentiality (e.g. the sole vulnerable component AKT in the caspase3-FKHR network has essentiality 1.0, and all vulnerable components in the p53 network have essentialities larger than 0.9 according to our ESM measure). In our study, we propose ESMs as the basic unit of signal transduction. In addition to the systematic evaluation of essentiality of signaling components done here, the concept of ESM opens new avenues of research relating the structure and function of signaling networks, as discussed above.

The network expansion method proposed here has a potential limitation in handling overall activating input-output paths that have inhibitory edges separated by more than one activating edge. Such paths of the original network may be broken in the expanded network, because we introduce complementary nodes only for the nodes with direct inhibitory roles. If the nodes situated between the first (third, ...) and second (fourth, ...) inhibitory edge in the overall activating path already have complementary nodes in the expanded network due to their involvement in other paths, the path will be retained in the expanded network. If some of these intermediate nodes do not have complementary nodes, but these nodes are involved in other input-output paths, their importance may be somewhat underestimated. If the intermediate nodes are not involved in other paths, their essentiality may be seriously underestimated. A potential solution to this problem is to add a step in the network expansion procedure: after introducing complementary nodes for all nodes with direct inhibitory effects, we enumerate all activating input-output paths with inhibitory edges separated by more than one activating edge and introduce the complementary nodes necessary for the maintenance of these paths in the extended network. The edges of these complementary nodes are determined from the negation of the Boolean rules in which the original nodes participate in. The tradeoff of completeness is the increase in size and redundancy of the expanded network. The signalling networks evaluated in this study have no, one and two instances, respectively, of a pair of inhibitory edges separated by more than one activating edge, and applying the solution described above has negligibly minor effects on the results. Given the density of feedforward and feedback loops in signalling networks, and the propensity for direct "inhibit the inhibitor" structures [49,50], we expect that our choice to focus on direct inhibitory effects is the more practical to make.

The aim of graph theoretical analysis of signaling networks is to provide primary clues for a better understanding of the signal transduction process [51]. For example, graph analysis of a large mammalian neuronal cell signaling network [14] revealed a separation of positive and negative feedback loops based on their graph distance from signals, suggesting an architecture that promotes dynamic stability and allows signals to persist. The shortest positive or negative paths among pairs of nodes can be used to determine a dependency matrix [10,11,32,35] which reflects the long-range regulatory relationships among signaling components. The method proposed here augments graph theory and allows it to address important functional aspects of signaling components, leading to testable predictions of comparable accuracy as dynamic models.

## Conclusions

Quantitative dynamic modelling of signaling networks helps to understand complex signal transduction processes, but it depends heavily on known mechanistic details and kinetic parameters. At the same time, structural analysis is emerging as a feasible and useful alternative for interpreting signal transduction. Aiming to overcome the limitations of existing structure-based approaches, we present an integrative computational method for evaluating the essentiality of components in signaling networks. The main steps of our method are expanding an existing signaling network to a richer representation that incorporates the positive or negative nature of interactions and the synergistic behaviors among multiple components and ranking the importance of signaling components by the cascading effects of their perturbation on the elementary signaling modes of the network. Validation on several signaling networks shows that this method can effectively uncover the essentiality of components mediating a signal transduction process. We conclude that while some properties of a dynamic model may depend on initial conditions and update time scales, other properties are encoded in the combinatorial regulations represented by Boolean rules and do not depend on the details of the dynamic simulation. Our method distils the most important ingredients of a dynamic model and integrates them into the network topology without the necessity of dynamic simulation. This method can be effectively used for exploratory analysis of large signaling networks where dynamic modeling or experimental tests are impractical and its results can guide targeted computational or experimental design.

## Methods

### Synthesizing evidence for inhibition and synergy

If the inhibitory regulations and combinatorial regulations in a signaling network are known, as was the case in [30-33,35,39], we can directly use them. Notably our method benefits even from partial information of inhibitory regulations and synergistic interactions. We introduce a parsimonious logical description of the activation status of each node. Utilizing the biological information collected from the literature (e.g. knockout studies), we employ the logical operators OR and AND to distinguish between independent and conditionally dependent interactions. In the absence of evidence for synergy, the default representation of multiple activating edges converging on the same node is an OR relationship. Information on conditional dependence can be readily incorporated by AND relationships among edges. Inhibitory regulations are represented by the logical operator NOT. If an inhibitory regulation is dominant among multiple interactions, as is often the case [48,52], we use AND NOT; otherwise we use OR NOT, which means that the absence of an inhibitor is similar to the presence of an activator. In this way our method works even if no or little information on conditional dependence is available, and can be iteratively improved as new information becomes available.

### Determining cascading effects of component disruption

Given an expanded signaling network *G *= (*V, E*), where *V *is the set of signaling components, and *E *is the set of regulatory interactions, we determine the cascading effect of the removal of a node by an iterative algorithm as follows:

• Step 1. Remove an original node or a complementary node *v *from the expanded signaling network. All the interactions starting or ending at *v *disappear accordingly.

• Step 2. For each direct target of *v*, say *u*, examine if its regulation by *v *is indispensable for its activation. If so, we store *u *into a set *K*, indicating that the removal of *v *leads to the disruption or inactivation of *u*.

• Step 3. Take a node *u *from *K*. Repeat Step 1 and Step 2 until *K *becomes empty.

We need to check at most each node and each edge of the expanded signaling network to determine the cascading effects of the removal of a node. Therefore, the worst time complexity of the iterative algorithm is *O*(|*E*|), where |*E*| is the number of the edges in *G*.

### Shortest elementary signaling modes

Multiple edges ending at a composite node in a signaling network are conditionally dependent and the activation of this node requires the activation of all its regulators. Thus, a composite node's activation follows the regulator that is activated last. In contrast, the activation of an original node or a complementary node can be done by any of several independent regulators and thus follows the regulator activated first. If we use the distance of signaling components from the input as a proxy for the sequence of events in signal transduction, the distance from the input node to a node *v *can be defined as:

where *A *is the adjacency matrix of the expanded network. We use a dynamic programming algorithm to determine the distance between an input node and output node which also detects shortest ESMs (Additional file 1).

### Essentiality of signaling components

Elementary signaling modes (ESMs) can be used to define an importance measure for the essentiality of signaling components in two different ways. First, we can rank the effect of a node's removal on the length of the shortest ESM. Second, we can determine the reduction in the number of ESMs following the removal of a node *v*:

where *N*_ESM_(*G*) and *N*_ESM_(*G_Δv_*) denote the total number of ESMs from the input(s) to the output(s) in the original expanded network *G *and the damaged network *G_Δv _*after deleting *v*, respectively. This ESM measure takes values in the interval [0,1], with 1 indicating a node whose loss disrupts all ESMs between the input and output node(s).

We also consider a more traditional graph measure for the essentiality of signaling components based on the number of all simple paths (SPs) from inputs to outputs:

where *N*_SP_(*G*) and *N*_SP_(*G_Δv_*) denote the total number of simple paths from the input(s) to the output(s) in the original expanded network *G *and the damaged network *G_Δv _*after deleting *v*, respectively. This SP measure also takes values in the interval [0,1], with 1 indicating a node whose loss causes the disruption of all paths between the input and output node(s). There are polynomial algorithms for computing the paths between any pair of nodes in a graph [53]. Here we are only interested in the paths between two specific nodes, so we adapt the depth-first search algorithm in graph theory to efficiently compute all the simple paths between the signal input and the response output (Additional File 1).

### Implementation of the method

All the algorithms in this study were coded and implemented in Matlab 7.6 (The Mathworks, Inc.). The depth-first algorithms are the Matlab implementation of the pseudo code given in the Additional file 1. The ILP problem is solved by using the command bintprog in the Matlab Optimization Toolbox which uses a branch-and-bound algorithm to solve binary integer linear programming problems. Programs are available at http://www.phys.psu.edu/~ralbert/ESM.

## Authors' contributions

RSW and RA conceived and designed the study. RSW performed the study. RSW and RA analyzed the results. RSW and RA wrote, read, and approved the final manuscript.

## Supplementary Material

Additional file 1 **Algorithms developed and used in this study**. This file contains the algorithms developed and used in this study, including the depth-first-search algorithm for enumerating all simple paths from the input node(s) to the output node(s) of a signaling network, the iterative integer linear programming algorithm for enumerating elementary signaling modes, the depth-first-search algorithm for estimating the number of elementary signaling modes, as well as the dynamic programming algorithm for finding shortest elementary signaling modes. Click here for file

Additional file 2 **Essentiality of the components from dynamic models of the three signaling networks**. This file describes the essentiality of the signaling components obtained by dynamic simulation of Boolean models for the host immune response network and the guard cell ABA signaling network, and logical steady state analysis of the T cell receptor signaling network (Tables S1-S4). Click here for file

Additional file 3 **Essentiality of the guard cell ABA signaling components from our method**. This file contains the importance values of the guard cell ABA signaling components obtained by single-node deletions (Figure S1) and two-node deletions (Figure S2), and literature support of the uncovered essential components. Click here for file

Additional file 4 **The expanded T cell receptor signaling network**. This file contains the expanded T cell receptor signaling network (Figure S3) and the importance values of the T cell receptor signaling components found by our method with AP as the input node (Figure S4). Click here for file

## References

[B1] Barabasi AL Oltvai ZN Network biology: understanding the cell's functional organization Nat Rev Genet 2004 5 2 101 113 10.1038/nrg1272 14735121

[B2] Albert R Scale-free networks in cell biology J Cell Sci 2005 118 Pt 21 4947 4957 10.1242/jcs.02714 16254242

[B3] Lee TI Rinaldi NJ Robert F Odom DT Bar-Joseph Z Gerber GK Hannett NM Harbison CT Thompson CM Simon I Transcriptional regulatory networks in Saccharomyces cerevisiae Science 2002 298 5594 799 804 10.1126/science.1075090 12399584

[B4] Jeong H Mason SP Barabasi AL Oltvai ZN Lethality and centrality in protein networks Nature 2001 411 6833 41 42 10.1038/35075138 11333967

[B5] Jeong H Tombor B Albert R Oltvai ZN Barabasi AL The large-scale organization of metabolic networks Nature 2000 407 6804 651 654 10.1038/35036627 11034217

[B6] Albert R Wang RS Discrete dynamic modeling of cellular signaling networks Methods Enzymol 2009 467 281 306 full_text 19897097 10.1016/S0076-6879(09)67011-7

[B7] Albert R Jeong H Barabasi AL Error and attack tolerance of complex networks Nature 2000 406 6794 378 382 10.1038/35019019 10935628

[B8] Stelling J Klamt S Bettenbrock K Schuster S Gilles ED Metabolic network structure determines key aspects of functionality and regulation Nature 2002 420 6912 190 193 10.1038/nature01166 12432396

[B9] Palumbo MC Colosimo A Giuliani A Farina L Functional essentiality from topology features in metabolic networks: a case study in yeast FEBS Lett 2005 579 21 4642 4646 10.1016/j.febslet.2005.07.033 16095595

[B10] Klamt S Saez-Rodriguez J Lindquist JA Simeoni L Gilles ED A methodology for the structural and functional analysis of signaling and regulatory networks BMC Bioinformatics 2006 7 56 10.1186/1471-2105-7-56 16464248 PMC1458363

[B11] Klamt S Saez-Rodriguez J Gilles ED Structural and functional analysis of cellular networks with CellNetAnalyzer BMC Syst Biol 2007 1 2 10.1186/1752-0509-1-2 17408509 PMC1847467

[B12] Goemann B Wingender E Potapov AP An approach to evaluate the topological significance of motifs and other patterns in regulatory networks BMC Syst Biol 2009 3 53 10.1186/1752-0509-3-53 19454001 PMC2694767

[B13] Wunderlich Z Mirny LA Using the topology of metabolic networks to predict viability of mutant strains Biophys J 2006 91 6 2304 2311 10.1529/biophysj.105.080572 16782788 PMC1557581

[B14] Ma'ayan A Jenkins SL Neves S Hasseldine A Grace E Dubin-Thaler B Eungdamrong NJ Weng G Ram PT Rice JJ Formation of regulatory patterns during signal propagation in a Mammalian cellular network Science 2005 309 5737 1078 1083 16099987 10.1126/science.1108876 PMC3032439

[B15] Binder B Ebenhoh O Hashimoto K Heinrich R Expansion of signal transduction networks Syst Biol (Stevenage) 2006 153 5 364 368 16986318 10.1049/ip-syb:20060030

[B16] Wasserman S Faust K Social Network Analysis: Methods and Applications 1994 Cambridge University Press, Cambridge, UK

[B17] Latora V Marchiori M A measure of centrality based on network efficiency New Journal of Physics 2007 9 188 10.1088/1367-2630/9/6/188

[B18] Freeman LC A set of measures of centrality based on betweenness Sociometry 1977 40 35 40 10.2307/3033543

[B19] Newman MEJ A measure of betweenness centrality based on random walks Social networks 2005 27 39 54 10.1016/j.socnet.2004.11.009

[B20] Bonacich P Some unique properties of eigenvector centrality Social Networks 2007 29 4 555 564 10.1016/j.socnet.2007.04.002

[B21] Perra N Fortunato S Spectral centrality measures in complex networks Phys Rev E Stat Nonlin Soft Matter Phys 2008 78 3 Pt 2 036107 10.1103/PhysRevE.78.036107 18851105

[B22] Crofts JJ Higham DJ A weighted communicability measure applied to complex brain networks J R Soc Interface 2009 6 33 411 414 19141429 10.1098/rsif.2008.0484 PMC2658663

[B23] Yu H Greenbaum D Xin Lu H Zhu X Gerstein M Genomic analysis of essentiality within protein networks Trends Genet 2004 20 6 227 231 10.1016/j.tig.2004.04.008 15145574

[B24] Samal A Singh S Giri V Krishna S Raghuram N Jain S Low degree metabolites explain essential reactions and enhance modularity in biological networks BMC Bioinformatics 2006 7 118 10.1186/1471-2105-7-118 16524470 PMC1434774

[B25] del Rio G Koschutzki D Coello G How to identify essential genes from molecular networks? BMC Syst Biol 2009 3 102 10.1186/1752-0509-3-102 19822021 PMC2765966

[B26] Tieri P Valensin S Latora V Castellani GC Marchiori M Remondini D Franceschi C Quantifying the relevance of different mediators in the human immune cell network Bioinformatics 2005 21 8 1639 1643 10.1093/bioinformatics/bti239 15613387

[B27] Potapov AP Goemann B Wingender E The pairwise disconnectivity index as a new metric for the topological analysis of regulatory networks BMC Bioinformatics 2008 9 227 10.1186/1471-2105-9-227 18454847 PMC2396639

[B28] Remenyi A Scholer HR Wilmanns M Combinatorial control of gene expression Nat Struct Mol Biol 2004 11 9 812 815 10.1038/nsmb820 15332082

[B29] Liu W Li D Zhang J Zhu Y He F SigFlux: a novel network feature to evaluate the importance of proteins in signal transduction networks BMC Bioinformatics 2006 7 515 10.1186/1471-2105-7-515 17129367 PMC1683949

[B30] Thakar J Pilione M Kirimanjeswara G Harvill ET Albert R Modeling systems-level regulation of host immune responses PLoS Comput Biol 2007 3 6 e109 10.1371/journal.pcbi.0030109 17559300 PMC1892604

[B31] Li S Assmann SM Albert R Predicting essential components of signal transduction networks: a dynamic model of guard cell abscisic acid signaling PLoS Biol 2006 4 10 e312 10.1371/journal.pbio.0040312 16968132 PMC1564158

[B32] Saez-Rodriguez J Simeoni L Lindquist JA Hemenway R Bommhardt U Arndt B Haus UU Weismantel R Gilles ED Klamt S A logical model provides insights into T cell receptor signaling PLoS Comput Biol 2007 3 8 e163 10.1371/journal.pcbi.0030163 17722974 PMC1950951

[B33] Zhang R Shah MV Yang J Nyland SB Liu X Yun JK Albert R Loughran TP Jr Network model of survival signaling in large granular lymphocyte leukemia Proc Natl Acad Sci USA 2008 105 42 16308 16313 10.1073/pnas.0806447105 18852469 PMC2571012

[B34] Albert R Othmer HG The topology of the regulatory interactions predicts the expression pattern of the segment polarity genes in Drosophila melanogaster J Theor Biol 2003 223 1 1 18 10.1016/S0022-5193(03)00035-3 12782112 PMC6388622

[B35] Samaga R Saez-Rodriguez J Alexopoulos LG Sorger PK Klamt S The logic of EGFR/ErbB signaling: theoretical properties and analysis of high-throughput data PLoS Comput Biol 2009 5 8 e1000438 10.1371/journal.pcbi.1000438 19662154 PMC2710522

[B36] Schuster S Fell DA Dandekar T A general definition of metabolic pathways useful for systematic organization and analysis of complex metabolic networks Nat Biotechnol 2000 18 3 326 332 10.1038/73786 10700151

[B37] Zevedei-Oancea I Schuster S A theoretical framework for detecting signal transfer routes in signalling networks Computers & Chemical Engineering 2005 29 596 617

[B38] Behre J Schuster S Modeling signal transduction in enzyme cascades with the concept of elementary flux modes J Comput Biol 2009 16 6 829 844 10.1089/cmb.2008.0177 19522666

[B39] Schlatter R Schmich K Avalos Vizcarra I Scheurich P Sauter T Borner C Ederer M Merfort I Sawodny O ON/OFF and beyond--a boolean model of apoptosis PLoS Comput Biol 2009 5 12 e1000595 10.1371/journal.pcbi.1000595 20011108 PMC2781112

[B40] Hornef MW Wick MJ Rhen M Normark S Bacterial strategies for overcoming host innate and adaptive immune responses Nat Immunol 2002 3 11 1033 1040 10.1038/ni1102-1033 12407412

[B41] Kirimanjeswara GS Mann PB Harvill ET Role of antibodies in immunity to Bordetella infections Infect Immun 2003 71 4 1719 1724 10.1128/IAI.71.4.1719-1724.2003 12654784 PMC152104

[B42] Harvill ET Cotter PA Miller JF Pregenomic comparative analysis between bordetella bronchiseptica RB50 and Bordetella pertussis tohama I in murine models of respiratory tract infection Infect Immun 1999 67 11 6109 6118 10531274 10.1128/iai.67.11.6109-6118.1999 PMC97000

[B43] Kindt TJ Osborne BA Goldsby RA W. H. Freeman Kuby Immunology 2006 6

[B44] Kwak JM Mäser P Schroeder JI Robert L The Clickable Guard Cell, Version II: Interactive Model of Guard Cell Signal Transduction Mechanisms and Pathways The Arabidopsis Book 2009 10.1199/tab.0114 PMC3243356 22303239

[B45] Zhong Q Simonis N Li QR Charloteaux B Heuze F Klitgord N Tam S Yu H Venkatesan K Mou D Edgetic perturbation models of human inherited disorders Mol Syst Biol 2009 5 321 10.1038/msb.2009.80 19888216 PMC2795474

[B46] Samaga R Von Kamp A Klamt S Computing combinatorial intervention strategies and failure modes in signaling networks J Comput Biol 2010 17 1 39 53 10.1089/cmb.2009.0121 20078396

[B47] Soni AS Jenkins JW Sundaram SS Determination of critical network interactions: an augmented Boolean pseudo-dynamics approach IET Syst Biol 2008 2 2 55 63 10.1049/iet-syb:20070025 18397116

[B48] Abdi A Tahoori MB Emamian ES Fault diagnosis engineering of digital circuits can identify vulnerable molecules in complex cellular pathways Sci Signal 2008 1 42 ra10 10.1126/scisignal.2000008 18941139

[B49] Ma'ayan A Lipshtat A Iyengar R Sontag ED Proximity of intracellular regulatory networks to monotone systems IET Syst Biol 2008 2 3 103 112 18537452 10.1049/iet-syb:20070036 PMC2453221

[B50] Gustafsson M Hornquist M Bjorkegren J Tegner J Genome-wide system analysis reveals stable yet flexible network dynamics in yeast IET Syst Biol 2009 3 4 219 228 10.1049/iet-syb.2008.0112 19640161

[B51] Ma'ayan A Insights into the organization of biochemical regulatory networks using graph theory analyses J Biol Chem 2009 284 9 5451 5455 18940806 10.1074/jbc.R800056200 PMC2645810

[B52] Wu Y Zhang X Yu J Ouyang Q Identification of a topological characteristic responsible for the biological robustness of regulatory networks PLoS Comput Biol 2009 5 7 e1000442 10.1371/journal.pcbi.1000442 19629157 PMC2704863

[B53] Rubin F Enumerating all simple paths in a graph IEEE Trans on Circuits and Systems 1978 25 8 641 642 10.1109/TCS.1978.1084515

