# Supplementary material for: Elementary signaling modes predict the essentiality of signal transduction network components
Source: BMC Syst Biol. 2011 Mar 22;5:44. doi: 10.1186/1752-0509-5-44 (PMC3070649; doi:10.1186/1752-0509-5-44)
Supplement: Additional file 1 — Algorithms developed and used in this study. This file contains the algorithms developed and used in this study, including the depth-first-search algorithm for enumerating all simple paths from the input node(s) to the output node(s) of a signaling network, the iterative integer linear programming algorithm for enumerating elementary signaling modes, the depth-first-search algorithm for estimating the number of elementary signaling modes, as well as the dynamic programming algorithm for finding shortest elementary signaling modes. [file 1752-0509-5-44-S1.PDF]

Additional file 1- Algorithms developed and used in this study

In this file we describe our depth-first search algorithm for enumerating all simple paths between two nodes, our integer linear programming algorithm for enumerating all elementary signaling modes (ESMs) or a certain number of ESMs, and our depth-first search algorithm for estimating the number of ESMs. The following notations are used:

V: the node set of an expanded signaling network *G*
E: the edge set of an expanded signaling network *G*
A: the adjacency matrix of an expanded signaling network *G*
R: the set of original nodes, which is a subset of *V*
C: the set of complementary nodes, which is a subset of *V*
S: the set of synergistic (composite) nodes, which is a subset of *V*
s: the input node of a signaling network
o: the output node of a signaling network

1. A depth-first search algorithm for enumerating all the paths between two nodes

Depth-first search (DFS) is an algorithm for traversing a graph. It starts at a source node and explores as far as possible along each branch (e.g. until a goal node or a leaf node is hit) before backtracking. When the search backtracks, it returns to the most recent node it has not finished exploring. Unlike the standard searching process of DFS where each branch is visited exactly once, the DFS for enumerating all simple paths between two nodes may visit a branch many times. A recursive version of DFS for enumerating all simple paths between two nodes, *s* and *o*, is as follows:

$\text{path} = [], \text{onpath} = [], \ p = 0$

$\text{DFS\_Path}(A, V, s, o, \text{path}, \text{onpath})$

1. $\text{path.append}(s), \text{onpath}(s) = 1$
2. $\text{if } (s = o)$
3. $\quad p = p + 1, \text{print path}$
4. $\text{else} \quad \text{for all } w \text{ in } V$
5. $\qquad \text{if } (A(s, w) = 1 \text{ and onpath}(w) = 0)$
6. $\qquad\quad \text{DFS\_Path}(A, w, o, \text{path}, \text{onpath})$
7. $\text{path.remove}(s), \text{onpath(s)} = 0$

In this algorithm, ‘path’ is a stack storing the current (partial) path from the input node. ‘onpath’ is a vector used to memorize whether a node is already included in the current path or not. *p* denotes the number of paths visited from the input node to the output node.

2. An integer linear programming algorithm to enumerating elementary signaling modes (ESMs)

We develop an integer linear programming (ILP) algorithm to enumerate ESMs. We search an ESM backward from the output node *o*. To activate the output node *o*, at least one of its regulators should be activated. If the regulator *u* is a composite (synergistic) node, then all the regulators of *u* are required to be activated; otherwise, the activation of one of its regulators is sufficient to activate *u*. The search continues until the input node *s* is reached. In this process, the objective is to find a minimal set of signaling components. We define binary variables for nodes, x_i={0,1}, and edges, $y_{i,j}$={0,1}, where 1 denotes that the node or edge is selected (activated) in the ESM; 0 means that the node or edge is not selected (inactive). The integer linear program for determining an ESM is formulated as follows:

$$
\begin{aligned}
\max \quad & wx_{out} - \sum_i x_i - \sum_{i,j} y_{i,j} \\
s.t. \quad & x_i \le y_{j,i} \quad \text{for all } v_i \in S \text{ and } A(j,i) = 1 \\
& x_k \le \sum\nolimits_s A(s,k) y_{s,k} \quad \text{for all } v_k \notin S \text{ and } \sum\nolimits_s A(s,k) \ge 1 \\
& y_{j,i} \le x_i \quad \text{for all } A(j,i) = 1 \\
& y_{j,i} \le x_j \quad \text{for all } A(j,i) = 1 \\
& x_i \in \{0,1\}, y_{i,j} \in \{0,1\}
\end{aligned}
$$

The first term in the objective function means that the ESM should be able to activate the output *o*: x_{out}=1, and the second term means that the ESM should have no redundant nodes and edges. The weight *w* is a large positive constant, indicating that the activation of the output has high priority and must be achieved. The first constraint implies that the activation of a synergistic (composite) node requires the activation of all of its regulators. In other words, if one of its regulators is inactive, then the synergistic node cannot be activated. The second constraint means that the activation of an original node or a complementary node requires the activation of at least one of its regulators. In other words, if all of its regulators are inactive, then the node cannot be activated. The third and fourth constraints represent the relationships between node variables and edge variables: the activation of a regulatory interaction requires the activation of both the regulator and the target node.

In order to enumerate all ESMs, we use an iterative procedure that at each step adds an additional constraint to avoid selecting from the ESMs that have already been found in the previous steps. Assuming that the solution associated with the ESM found in a previous step is [x_i^0, $y_{i,j}^0$], then the iterative procedure will add the following constraint to the formulated ILP in the last step:

$$\sum_i x_i^0 x_i + \sum_{i,j} y_{i,j}^0 y_{i,j} \le \sum_i x_i^0 + \sum_{i,j} y_{i,j}^0 - 1.$$

The advantage of this ILP-based iterative procedure is its ability to enumerate the *k* shortest ESMs (here 'short' is in terms of the number of nodes and edges, which means that the procedure iterates *k* steps and then stops). It can also find all ESMs with no more than a certain number of interactions or nodes by adding constraints as follows:

$$\sum_i x_i \le N \qquad \text{or} \qquad \sum_{i,j} y_{i,j} \le L$$

where N and L are the numbers of nodes and edges respectively in the ESMs. We are only interested in the ESMs located in the paths from the input node to the output node, and thus we constrain the variables for the nodes and edges not located in the input-output paths to zeros in a preprocessing step.

3. A depth-first-search algorithm for estimating the number of ESMs

We develop an efficient depth-first-search (DFS)-based algorithm for estimating the number of ESMs in a large signaling network. This algorithm searches the network from a source node s in a depth-first manner. When meeting an original node or a complementary node, it continues to search the lower branches of the network. When meeting a composite (synergistic) node, it checks if all the regulators of this composite node have been visited so far. If so, it will continue to search the lower branches; otherwise, it will turn back to upper branches. A non-recursive version of the algorithm is as follows:

```
DFS_ESM(A,V,s,o)
  Initialization
edgelist = stack(),  branch = vector(),  ESM_num = 0
for all v do  weight(v) = 1, vtimes_new(e_{·,v}) = 0, vtimes_old(e_{·,v}) = 0
  Visit the source node
for all v_i ∈ V and A(s, i) = 1
    branch(1) = s, edgelist.append(e_{s,i}),
  Visit the downstream branches
while(edgelist ≠ ϕ)
    e_{u,w} = edgelist.pop()
    if (v_w ∈ S)
        //Recording the visiting times for the possible combinations of ESMs
        vtimes_old = vtimes_new
        vtimes_new(e_{u,w}) = vtimes_new(e_{u,w}) + weight(v_u)
    branch = branch(1 : index(branch(:) = u)), branch.append(w)
    if(branch(:)! = w)
        if (w = o)  do  ESM_num = ESM_num + weight(u)
        else  if(v_w ∉ S or (v_w ∈ S and vtimes_new(e_{·,w}) > 0))
                                     //If v_w qualifies, the search continues
                for all v_j ∈ V and A(w, j) = 1
                    edgelist.append(e_{w,j})
                    if(v_w ∉ S)
                        weight(v_w) = weight(v_u)
                    if (v_w ∈ S)
                        weight(v_w) = ∏ vtimes_new(e_{·,w}) − ∏ vtimes_old(e_{·,w})
  Return the results
return ESM_num
```

In this algorithm, 'edgelist' is a stack storing the edges in the current (partial) elementary signaling mode (ESM). 'branch' is a vector used to memorize whether a node is already included in the current ESM or not. 'weight' is a node vector to record the combinations of (partial) ESMs occurring at a node. 'vtimes_new' and 'vtimes_old' are edge vectors to record the visiting times occurring at an edge to compute the combinations of (partial) ESMs. 'ESM_num' denotes the number of ESMs visited from the input node to the output node.

The main difference between this DFS algorithm and those used for searching general graphs lies in its operation on composite nodes (Step 7 and Step 18). It records the visiting times of each edge ending at a composite node for the possible combinations of ESMs. Only when all the edges ending at a composite node have been visited, can the algorithm continue to search the branches downstream of the composite node. Doing so, it transfers the possible combinations occurring at the composite node to the downstream nodes by the calculation in Step 17 and Step 19. For a large signaling network, the number of ESMs may be prohibitively large due to the combinations of simple paths upstream of the composite nodes. In such a case, different ESMs overlap with each other and lead to heavy redundancy. To sample main scaffolding ESMs, we can use the 'max' operation instead of the multiplier operation in Step 19. If the network has two input nodes, one should just store the information in 'weight', 'vtimes_new', 'vtimes_old', and 'ESM_num', and repeat the algorithm with the second input node.

4. A dynamic programming algorithm for determining shortest ESMs

Shortest paths from a purely topological view may not be biologically sufficient routes of signal transduction. Shortest ESMs (in terms of the distance between the input node and the output node) incorporate synergistic regulations and can reflect signal transduction more properly. We develop a dynamic programming (DP) algorithm to determine the shortest ESM and its length in the following way:

ShortestESM(A,V,s,o)

Initialization

1. $d[s]=0,\ p[s]=\text{NULL}$
2. for all $v\in V$
3. if $v\in R,C$ do $d[v]=+\infty,\ p[v]=\text{NULL}$
4 if $v\in S$ do $d[v]=-\infty,\ p[v]=\text{NULL}$
5. $P\leftarrow\phi,\ Q\leftarrow V$

Update distances iteratively

6. while $Q\neq\phi$
7. do $u\leftarrow \text{ExtractMin}(Q)$
8. $P\leftarrow P\bigcup\{u\}, Q\leftarrow Q\setminus\{u\}$
9. for each node v and $A(u,v)=1$
10. if ($v\in R,C$ and $d[v]>d[u]+1$)
11. $d[v]=d[u]+1,\ p[v]=u$
12. if ($v\in S$ and $d[v]<\text{d}[u]+1$)
13. $d[v]=d[u]+1,\ p[v]\leftarrow\{u\}$

Return the result

14. return $d[o],\ p(\cdot)$

Here $d[v]$ denotes the distance between s to v and $p[v]$ denotes the direct regulators (parents) of v in the shortest ESM. P represents the set of nodes which are already assigned a shortest distance from s. ExtractMin(Q) selects the node u with minimum $d[u]$ among all original or complementary nodes in the current node set Q and among composite nodes in Q whose regulators are all in P. The algorithm keeps selecting a node in Q through ExtractMin(Q) and adds it into P. When u enters into P, the distances of all its direct downstream nodes are updated accordingly. Finally, the algorithm returns the shortest distance from the input node s to the output node o. The corresponding shortest ESM can be retrieved from $p[o]$ in a backward way.
