# Supplementary material for: Elementary signaling modes predict the essentiality of signal transduction network components
Source: BMC Syst Biol. 2011 Mar 22;5:44. doi: 10.1186/1752-0509-5-44 (PMC3070649; doi:10.1186/1752-0509-5-44)
Supplement: Additional file 2 — Essentiality of the components from dynamic models of the three signaling networks. This file describes the essentiality of the signaling components obtained by dynamic simulation of Boolean models for the host immune response network and the guard cell ABA signaling network, and logical steady state analysis of the T cell receptor signaling network (Tables S1-S4). [file 1752-0509-5-44-S2.PDF]

Additional file 2- Essentiality of the components from dynamic models of the three signaling networks

This section contains the essentiality of signaling components obtained by dynamic simulation of Boolean models for the host immune response network and the guard cell ABA signaling network, and logical steady-state analysis of the T cell receptor signaling network. For the host immune response network and the guard cell ABA signaling network, the dynamic simulations were performed by the random order asynchronous Boolean models in Thakar *et al.* 2007 and Li *et al*. 2006 respectively, following the same specifications as originally described. Briefly, a timestep of a random order asynchronous model corresponds to a round of update in which a uniformly randomly selected permutation of the nodes is generated and the nodes' states are updated in that order.

As described in Thakar *et al.* 2007, biologically meaningful constraints to the update orders are used (e.g. phagocytosis is updated last, epithelial cells are updated before dendritic cells). These constraints yield a reproducible (update order independent) pattern of activity for all nodes. The initial condition is a state in which only bacteria are ON (1) and all the other nodes are OFF (0). The normal outcome of the host immune response model is bacterial clearance caused by sustained phagocytosis (i.e. the node PH=1) after around 20 timesteps. A component is classified as essential if given bacterial infection, Bt=1, perturbing this component affects the normal outcome (i.e the original model returns bacterial clearance, PH=1, and the perturbed model returns bacterial persistence, PH=0). Otherwise, the component is classified as non-essential.

For the guard cell ABA signaling network, we run the asynchronous Boolean dynamic model of Li *et al.* 2006 by using the software BooleanNet developed in Albert *et al.* 2008. The initial conditions are sampled uniformly randomly and a large number of replicate simulations are performed. The output of the model is the percentage of closed stomata (i.e. of the node Closure =1) in these replicate simulations, and the normal outcome is 100% closure within eight time steps. The classification of the outcomes follows those in Li *et al*. 2006: ABA insensitivity means that the probability (percentage) of closure is zero after the first three steps; reduced ABA sensitivity means that the probability of closure is always less than 100%; ABA hyposensitivity corresponds to ABA-induced closure that is slower than wild-type; ABA hypersensitivity corresponds to ABA-induced closure that is faster than wild-type. As in this study we focus on deleterious effects, we treat ABA hypersensitivity as wild type. The components whose perturbation leads to ABA insensitivity, reduced ABA sensitivity, or ABA hyposensitivity are classified as essential. The components whose perturbation leads to wild type are classified as non-essential.

For the T cell receptor signaling network, we perform the logical steady-state analysis of the logic model in Saez-Rodriguez *et al*. 2007 by using the software CellNetAnalyzer developed in Klamt *et al.* 2007. In the unperturbed network, the outputs NFκB and AP1 reach 1 in the steady state. The essentiality of a signaling component is defined by checking whether its perturbation affects the states of the outputs in the steady state. For all the three networks, knockout of a node is simulated by maintaining the node in the OFF state and constitutive activation of a node is simulated by maintaining the node in the ON state. The results of node knockouts will be compared to our method's results for deletion of original nodes in the

expanded network and the results of node constitutive activations will be compared to our method's results for deletion of complementary nodes in the expanded network.

The perturbation results of the Boolean dynamic model of the mammalian immune response network are shown in Table S1. According to the definition of essentiality described above, the nodes that are classified as essential are: Th2RC, DC, T0, AP, Th2C, BC, PIC, EC, ~Th1RC, ~Th2RC, and the nodes that are classified as non-essential are: AgAb, Cp, MP, RP, Oab, Th1RC, Th1C, Cab.

Table S1. Perturbation results of the Boolean dynamic model of the mammalian immune response network.

**Single-node knockout**			
**Component**	**Outcome**	**Component**	**Outcome**
Th2RC (Th2 related cytokines)	Bacterial persistence	AgAb (Ag-Ab complex)	Bacterial clearance
DC (Dendritic cells)	Bacterial persistence	Cp (Complement)	Bacterial clearance
T0 (T0 cells)	Bacterial persistence	MP (Macrophages)	Bacterial clearance
AP (Activated phagocytes)	Bacterial persistence	RP (Recruited PMNs)	Bacterial clearance
Th2C (T helper cell type 2)	Bacterial persistence	Oab (Other antibodies)	Bacterial clearance
BC (B cells)	Bacterial persistence	Th1RC (Th1 related cytokines)	Bacterial clearance
PIC (Proinflammatory cytokines)	Bacterial persistence	Th1C (T helper cell type 1)	Bacterial clearance
EC (Epithelial cells)	Bacterial persistence	Cab (Complement-fixing Abs)	Bacterial clearance
**Single-node constitutive activation**			
**Components**	**Outcome**	**Components**	**Outcome**
Th1RC	Bacterial persistence	Th2RC	Bacterial persistence

The perturbation results of the Boolean dynamic model of ABA signaling are shown in Table S2. According to the definition of essentiality described above, the nodes that are classified as essential are: Actin, Depolar, AnionEM, PLD, PA, AGB1, GPA1, S1P, SphK, pH_c, KOUT, Ca^{2+}_c, Atrboh, ROS, OST1, ROP2, ~Malate, ~RAC1, ~ABI1, ~Ca^{2+}ATPase, and the nodes that are classified as non-essential are: H^+ATPase, CaIM, KAP, ABI1, Ca^{2+}ATPase, CIS, PLC, cADPR, cGMP, InsP6, InsPK, InsP3, RCN, NOS, NIA12, NO, GC, ADPRc, KEV, ABH, ERA, ~Ca^{2+}_c, ~ROS, ~pH_c, ~KEV, ~Depolar, ~AnionEM, ~CIS, ~CaIM, ~H^+ATPase, ~ABH1, ~ERA1, ~PA, ~NO, ~KOUT, ~PEPC, ~GCR1.

Table S2. Perturbation results for the Boolean dynamic model of ABA signaling.

Single-node knockout			
**Component**	**Outcome**	**Components**	**Outcome**
Actin	ABA insensitivity	ABI1	Wild type
Depolar	ABA insensitivity	Ca^{2+}ATPase	Wild type
AnionEM	ABA insensitivity	CIS	Wild type
PLD	Reduced ABA sensitivity	PLC	Wild type
PA	Reduced ABA sensitivity	cADPR	Wild type
AGB1	Reduced ABA sensitivity	cGMP	Wild type
GPA1	Reduced ABA sensitivity	InsP6	Wild type
S1P	Reduced ABA sensitivity	InsPK	Wild type
SphK	Reduced ABA sensitivity	InsP3	Wild type
pH_c	Reduced ABA sensitivity	RCN1	Wild type
KOUT	Reduced ABA sensitivity	NOS	Wild type
Ca^{2+}_c	ABA hyposensitivity	NIA12	Wild type
Atrboh	ABA hyposensitivity	NO	Wild type
ROS	ABA hyposensitivity	GC	Wild type
OST1	ABA hyposensitivity	ADPRc	Wild type
ROP2	ABA hyposensitivity	KEV	Wild type
H^+ATPase	Wild type	ABH1	Wild type
CaIM	Wild type	ERA1	Wild type
KAP	Wild type		
**Single-node constitutive activation**			
**Components**	**Outcome**	**Components**	**Outcome**
Malate	ABA insensitivity	CIS	Wild type
RAC1	Reduced ABA sensitivity	CaIM	Wild type
ABI1	Reduced ABA sensitivity	H^+ATPase	Wild type
Ca^{2+}ATPase	ABA hyposensitivity	ABH1	Wild type
Ca^{2+}_c	Wild type	ERA1	Wild type
ROS	Wild type	PA	Wild type
pH_c	Wild type	NO	Wild type
KEV	Wild type	KOUT	Wild type
Depolar	Wild type	PEPC	Wild type
AnionEM	Wild type	GCR1	Wild type

The perturbation results by logical steady state analysis of the T cell receptor signaling network with NFκB as the output are shown in Table S3. According to the definition of essentiality described above, the nodes that are classified as essential to the output NFκB are: IKKab, Zap70, PLCga, TCRb, PKCth, IKKg, TCRp, DAG, Vav1, CaM, Ca, CaMK2, IP3, LAT, Abl, CARD11a, Fyn, SLP76, PIP3, PI3K, Gads, PLCgb, PDK1, ~DGK , ~Cblb, ~cCblp1, ~Gab2, ~IKB, ~cCblp2, ~PTEN , ~SHIP-1. The nodes that are

classified as non-essential to the output NFκB are: CD45, Lckp, CD4, Csk, PAG, Raf, Ras, ERK, RasGRP1, Grb2, Sos, MEK, x, sh3bp2, Rlk, cCblp1, cCblp2, vav3, Gab2, SHP1, Itk, Lckp2, HPK1, MEKK1, Rac1p1, Rac1p2, DGK, Cdc42, MLK3, ~GAPs, ~Lckp1, ~SHP1,~TCRlig, ~ERK, ~Csk, ~CD45, ~CD4, ~TCRb, ~FYN, ~PAG.

Table S3. Perturbation results of the logical steady state analysis of the T cell receptor signaling network with NFκB as the output. CD28 and TCRlig are inputs, and NFκB is the output. The nodes that obviously have no relevance to NFκB are not included in the table. These nodes are SRE, P38, MKK4, JNK, JUN, Fos, AP1, Rsk, CREB, CRE, NFAT, Calcin, CaMK4, p70s, SHP2, PKB for single-node knockout and Cabin, AKAP79, Calpr1, Gadd45 for constitutive activation.

**Single-node knockout**					
**Component**	**Outcome**	**Component**	**Outcome**	**Component**	**Outcome**
IKKab	NFκB = 0	PIP3	NFκB = 0	sh3bp2	NFκB = 1
Zap70	NFκB = 0	PI3K	NFκB = 0	Rlk	NFκB = 1
PLCga	NFκB = 0	Gads	NFκB = 0	cCblp1	NFκB = 1
TCRb	NFκB = 0	PLCgb	NFκB = 0	cCblp2	NFκB = 1
PKCth	NFκB = 0	PDK1	NFκB = 0	vav3	NFκB = 1
IKKg	NFκB = 0	CD45	NFκB = 1	Gab2	NFκB = 1
TCRp	NFκB = 0	Lckp1	NFκB = 1	SHP1	NFκB = 1
DAG	NFκB = 0	CD4	NFκB = 1	Itk	NFκB = 1
Vav1	NFκB = 0	Csk	NFκB = 1	Lckp2	NFκB = 1
CaM	NFκB = 0	PAG	NFκB = 1	HPK1	NFκB = 1
Ca	NFκB = 0	Raf	NFκB = 1	MEKK1	NFκB = 1
CaMK2	NFκB = 0	Ras	NFκB = 1	Rac1p1	NFκB = 1
IP3	NFκB = 0	ERK	NFκB = 1	Rac1p2	NFκB = 1
LAT	NFκB = 0	RasGRP1	NFκB = 1	DGK	NFκB = 1
Abl	NFκB = 0	Grb2	NFκB = 1	Cdc42	NFκB = 1
CARD11a	NFκB = 0	Sos	NFκB = 1	MLK3	NFκB = 1
Fyn	NFκB = 0	MEK	NFκB = 1		
SLP76	NFκB = 0	x	NFκB = 1		
**Single-node constitutive activation**					
**Component**	**Outcome**	**Component**	**Outcome**	**Component**	**Outcome**
DGK	NFκB = 0	SHIP-1	NFκB = 0	CD45	NFκB = 1
Cblb	NFκB = 0	GAPs	NFκB = 1	CD4	NFκB = 1
cCblp1	NFκB = 0	Lckp1	NFκB = 1	TCRb	NFκB = 1
Gab2	NFκB = 0	SHP1	NFκB = 1	FYN	NFκB = 1
IKB	NFκB = 0	TCRlig	NFκB = 1	PAG	NFκB = 1
cCblp2	NFκB = 0	ERK	NFκB = 1		
PTEN	NFκB = 0	Csk	NFκB = 1		

The perturbation results by logical steady state analysis of the T cell receptor signaling network with AP1 as the output are shown in Table S4. According to the definition of essentiality described above, the nodes that are classified as essential to the output AP1 are: ERK, Zap70, PLCga, TCRb, Vav1, MEK, TCRp, DAG, JUN, Ras, Raf, JNK, Fos, LAT, Abl, SLP76, PIP3, PI3K, Gads, PLCgb, Sos, RasGRP1, Grb2, Fyn, ~DGK, ~Cblb, ~cCblp1, ~Gab2, ~GAPs, ~cCblp2, ~PTEN, ~SHIP-1. The nodes that are classified as non-essential to the output AP1 are: Csk, PAG, x, MKK4, Lckp1, CD4, CD45, MLK3, sh3bp2, Rlk, Itk c, Cblp1, cCblp2, vav3, SHP1, Gab2, Lckp2, HPK1, MEKK1, Rac1p1, Rac1p2, DGK, Cdc42, ~Lckp1, ~SHP1, ~TCRlig, ~TCRb, ~ERK, ~Csk, ~CD45, ~CD4, ~FYN, ~PAG.

Table S4. Perturbation results of the logical steady state analysis of the T cell receptor signaling network with AP1 as the output. CD28 and TCRlig are inputs, and AP1 is the output. The nodes that obviously have no relevance to the output AP1 are not included in the table. These nodes are SRE, P38, Rsk, CREB, CRE, NFAT, Ca, CaM, CaMK2,CARD11a, PKCth, IKKg, IKKab, NFKB, Calcin, CaMK4, p70s, SHP2, PKB, PDK1, IP3 for single-node knockout and Cabin, AKAP79, Calpr1, Gadd45, IKB for constitutive activation.

**Single-node knockout**					
**Component**	**Outcome**	**Component**	**Outcome**	**Component**	**Outcome**
ERK	AP1=0	PIP3	AP1=0	sh3bp2	AP1=1
Zap70	AP1=0	PI3K	AP1=0	Rlk	AP1=1
PLCga	AP1=0	Gads	AP1=0	Itk	AP1=1
TCRb	AP1=0	PLCgb	AP1=0	cCblp1	AP1=1
Vav1	AP1=0	Sos	AP1=0	cCblp2	AP1=1
MEK	AP1=0	RasGRP1	AP1=0	vav3	AP1=1
TCRp	AP1=0	Grb2	AP1=0	SHP1	AP1=1
DAG	AP1=0	Fyn	AP1=0	Gab2	AP1=1
JUN	AP1=0	Csk	AP1=1	Lckp2	AP1=1
Ras	AP1=0	PAG	AP1=1	HPK1	AP1=1
Raf	AP1=0	x	AP1=1	MEKK1	AP1=1
JNK	AP1=0	MKK4	AP1=1	Rac1p1	AP1=1
Fos	AP1=0	Lckp1	AP1=1	Rac1p2	AP1=1
LAT	AP1=0	CD4	AP1=1	DGK	AP1=1
Abl	AP1=0	CD45	AP1=1	Cdc42	AP1=1
SLP76	AP1=0	MLK3	AP1=1		
**Single-node constitutive activation**					
**Component**	**Outcome**	**Component**	**Outcome**	**Component**	**Outcome**
DGK	AP1=0	PTEN	AP1=0	ERK	AP1=1
Cblb	AP1=0	SHIP-1	AP1=0	Csk	AP1=1
cCblp1	AP1=0	Lckp1	AP1=1	CD45	AP1=1
Gab2	AP1=0	SHP1	AP1=1	CD4	AP1=1
GAPs	AP1=0	TCRlig	AP1=1	FYN	AP1=1
cCblp2	AP1=0	TCRb	AP1=1	PAG	AP1=1

References

Albert I, Thakar J, Li S, Zhang R, Albert R: **Boolean network simulations for life scientists**. *Source Code Biol Med* 2008, **3**:16.

Li S, Assmann SM, Albert R: **Predicting essential components of signal transduction networks: a dynamic model of guard cell abscisic acid signaling**. *PLoS Biol* 2006, **4**(10):e312.

Klamt S, Saez-Rodriguez J, Gilles ED: **Structural and functional analysis of cellular networks with CellNetAnalyzer**. *BMC Syst Biol* 2007, **1**:2.

Saez-Rodriguez J, Simeoni L, Lindquist JA, Hemenway R, Bommhardt U, Arndt B, Haus UU, Weismantel R, Gilles ED, Klamt S *et al*: **A logical model provides insights into T cell receptor signaling**. *PLoS Comput Biol* 2007, **3**(8):e163.

Thakar J, Pilione M, Kirimanjeswara G, Harvill ET, Albert R: **Modeling systems-level regulation of host immune responses**. *PLoS Comput Biol* 2007, **3**(6):e109.
