# Supplementary material for: Elementary signaling modes predict the essentiality of signal transduction network components
Source: BMC Syst Biol. 2011 Mar 22;5:44. doi: 10.1186/1752-0509-5-44 (PMC3070649; doi:10.1186/1752-0509-5-44)
Supplement: Additional file 3 — Essentiality of the guard cell ABA signaling components from our method. This file contains the importance values of the guard cell ABA signaling components obtained by single-node deletions (Figure S1) and two-node deletions (Figure S2), and literature support of the uncovered essential components. [file 1752-0509-5-44-S3.PDF]

Additional file 3- Essentiality of guard cell ABA signaling components from our method

We first evaluate the importance of the activation of signaling components (i.e. real nodes) by considering single-node deletion of original nodes. The computational results are summarized in Figure S1(a). Our method shows that knockout of AnionEM, Depolar or Actin will completely block all ABA signaling paths and elementary signaling modes (ESMs), and disruption of other components such as GPA1, AGB1, CaIM, PLD, PA, pH_c, H^+ATPase, Ca^{2+}_c, or KOUT leads to a strong reduction of the signal transduction connectivity. The important components suggested by our method have been well documented in the literature. The dynamic model of Li *et al*. 2006 has shown that the loss of membrane depolarizability (Depolar), the disruption of anion efflux (AnionEM), and the loss of actin cytoskeleton reorganization (Actin) leads to ABA insensitivity (Li *et al*. 2006), which are also supported by experimental observations (Schwartz *et al*. 1995). Both dynamic models and experimental results have shown that single-node disruption of PLD, PA, SphK, S1P, GPA1, KOUT, and pH_c increase reduces ABA sensitivity (Li *et al*. 2006; Coursol *et al*. 2003). Blocking Ca^{2+}_c increase causes slower than wild-type ABA-induced closure (Li *et al*. 2006). In addition, the effect of disrupting Atrboh or ROS production is very similar to the effect of blocking Ca^{2+}_c increase in the dynamic model (Li *et al*. 2006). The main difference between the results given by the ESM measure and the SP measure lies in the higher importance of S1P and SphK given by the former, which is supported by the experimental observation that S1P and SphK are important for guard cell ABA-induce stomatal closure (Wang *et al*. 2001) and the dynamic simulation that disruption of S1P or SphK leads to ABA reduced sensitivity (Li *et al*. 2006). From these results we can see that our method is able to effectively capture the essentiality of signaling components. While several nodes identified as important components by our method also have high betweenness centrality, well documented important signaling components, such as Actin, pH_c, H^+ATPase, AGB1, GPA1, OST1 are not identified as important by betweenness centrality and *SigFlux*. This indicates that without incorporating synergistic and inhibitory regulations, the essentiality of some signaling components cannot be captured.

Next we consider single-node deletion of complementary nodes, which evaluates the importance of inhibitory regulation by the corresponding signaling components. The importance values based on the ESM measure and the SP measure are shown in Figure S1(b). Our method shows that knocking out the complementary node of Malate will completely block all signaling paths and ESMs for ABA signal transduction, indicating that the inhibition of Malate is essential for ABA signal transduction. We observe that some components whose activations are identified as important in Figure S1(a) have very high importance values in Figure S1(b), indicating the inhibitory regulations of these components are equally important, e.g. Ca^{2+}_c, Depolar, AnionEM, pH_c. Conversely, some components whose activating effects are not essential have large inhibitory effects, e.g. Ca^{2+}ATPase, KEV, ABI1. Indeed, the inhibitory regulations of ABI1 and Ca^{2+}ATPase have been revealed to significantly affect ABA-induced stomatal

closure (Li *et al*. 2006; Gosti *et al.* 1999). In addition, deletion of the complementary node of ABI1, H^{+}ATPase or RAC1 affects the length of shortest ESMs, suggesting that the inhibitions of these signaling components are relevant to the signal transduction efficiency. Again, betweenness centrality and the *SigFlux* measure cannot fully capture the importance of some inhibitors, e.g. Malate, Ca^{2+}ATPase, ROS, KEV, pH_c.

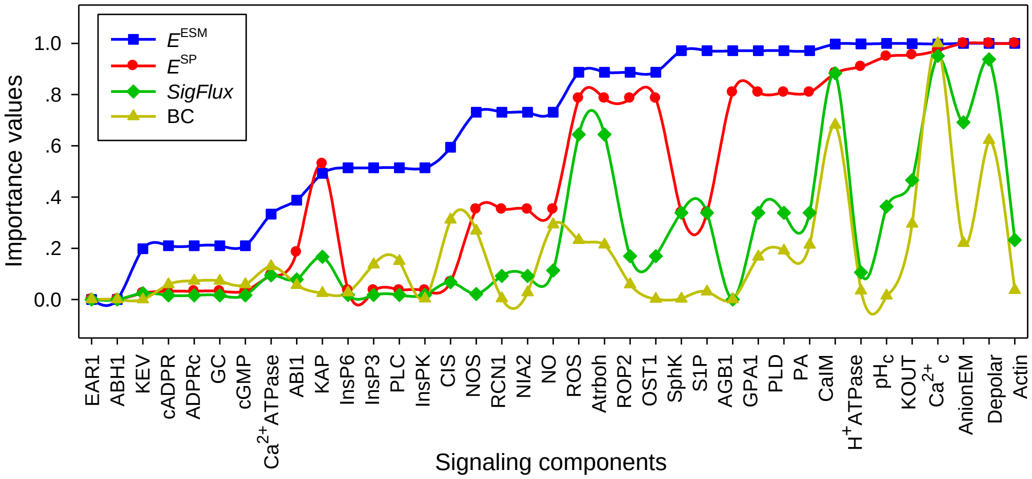

(a)

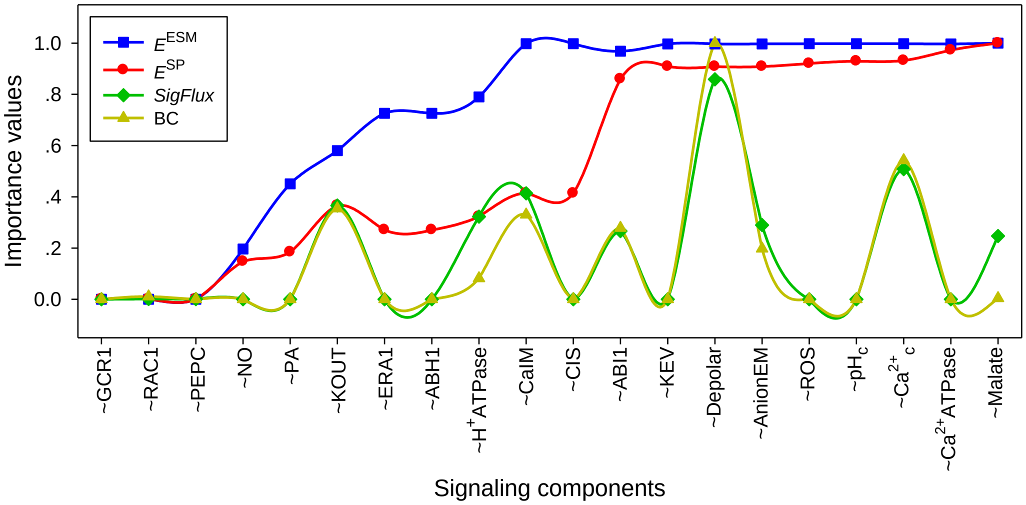

(b)

Figure S1 Comparison of different methods on the guard cell ABA signaling network (a) Importance values obtained by single-node deletions of original nodes. (b) Importance values obtained by single-nodes deletion of complementary nodes. Rectangles indicate the importance values or prediction accuracy obtained by the ESM measure, circles represent the simple path measure, diamonds denote the *SigFlux* measure, and triangles show the betweenness centrality measure.

We also consider the importance of two-node combinations by deleting two nodes simultaneously. The results indicate that two-node disruption of KAP and KOUT, pH_c and $Ca^{2+}{}_c$, ~ABI1 and ~Ca^{2+}ATPase, ~Ca^{2+}ATPase and ~RAC1 will completely disrupt the ABA signal transduction. All of these are consistent with the dynamic simulation result that combined perturbation of these components leads to ABA insensitivity (Li *et al*. 2006). The distribution of the importance values of all two-node combinations excluding those having the same importance values as their individual components is shown in Figure S2. We can see that there is a large fraction of two-node combinations whose perturbations lead to severe damage to ABA signal transduction. This is consistent with the dynamic simulation result in Li *et al*. 2006 that 55% of two-node disruptions lead to insensitivity, reduced sensitivity or hyposensitivity to the ABA signal. However, without considering the cascading effects of nodes (e.g. *SigFlux*), the consequences of two-node disruptions are heavily underestimated.

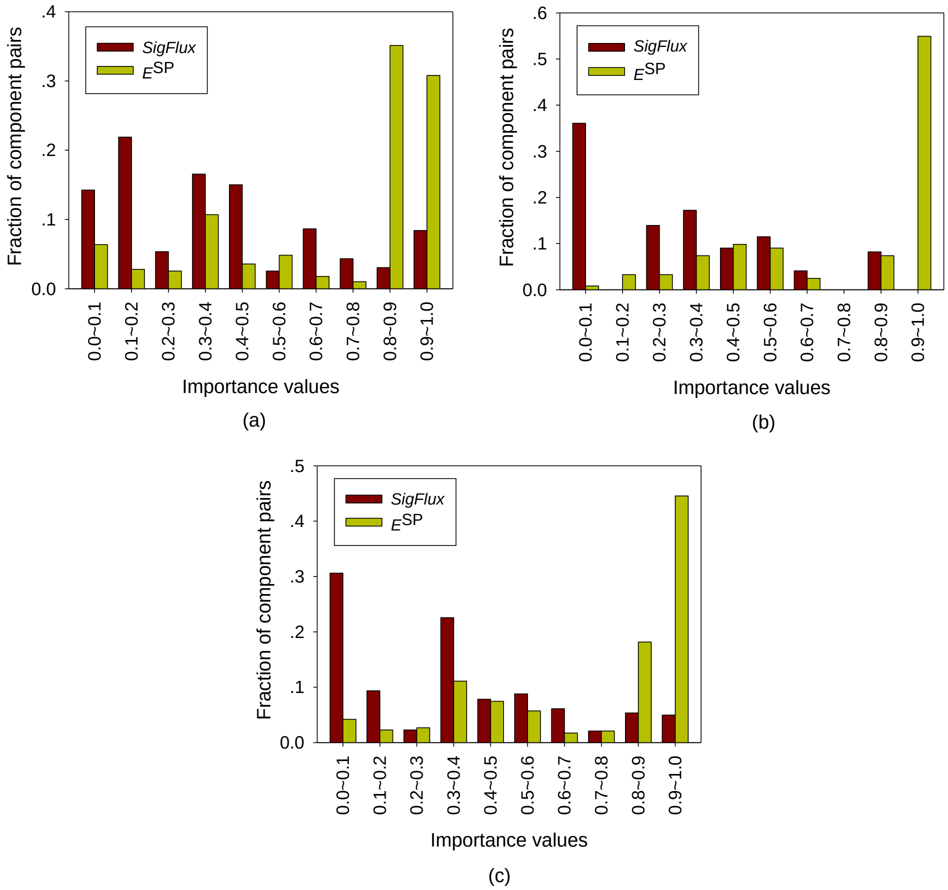

Figure S2. Distribution of importance values of two-node combinations. (a) Deletion of two original nodes. (b) Deletion of two complementary nodes. (c) Deletion of an original node and a complementary node.

References

Coursol S, Fan LM, Le Stunff H, Spiegel S, Gilroy S, Assmann SM: **Sphingolipid signalling in Arabidopsis guard cells involves heterotrimeric G proteins**. *Nature* 2003, **423**(6940):651-654.

Gosti F, Beaudoin N, Serizet C, Webb AA, Vartanian N, Giraudat J: **ABI1 protein phosphatase 2C is a negative regulator of abscisic acid signaling**. *Plant Cell* 1999, **11**(10):1897-1910.

Irving HR, Gehring CA, Parish RW: **Changes in cytosolic pH and calcium of guard cells precede stomatal movements**. *Proc Natl Acad Sci U S A* 1992, **89**(5):1790-1794.

Li S, Assmann SM, Albert R: **Predicting essential components of signal transduction networks: a dynamic model of guard cell abscisic acid signaling**. *PLoS Biol* 2006, **4**(10):e312.

Schwartz A, Ilan N, Schwarz M, Scheaffer J, Assmann SM, Schroeder JI: **Anion-Channel Blockers Inhibit S-Type Anion Channels and Abscisic Acid Responses in Guard Cells**. *Plant Physiol* 1995, **109**(2):651-658.

Wang XQ, Ullah H, Jones AM, Assmann SM: **G protein regulation of ion channels and abscisic acid signaling in Arabidopsis guard cells**. *Science* 2001, **292**(5524):2070-2072.
