# Supplementary material for: Elementary signaling modes predict the essentiality of signal transduction network components
Source: BMC Syst Biol. 2011 Mar 22;5:44. doi: 10.1186/1752-0509-5-44 (PMC3070649; doi:10.1186/1752-0509-5-44)
Supplement: Additional file 4 — The expanded T cell receptor signaling network. This file contains the expanded T cell receptor signaling network (Figure S3) and the importance values of the T cell receptor signaling components found by our method with AP as the input node (Figure S4). [file 1752-0509-5-44-S4.PDF]

Additional file 4- The expanded T cell receptor signaling network

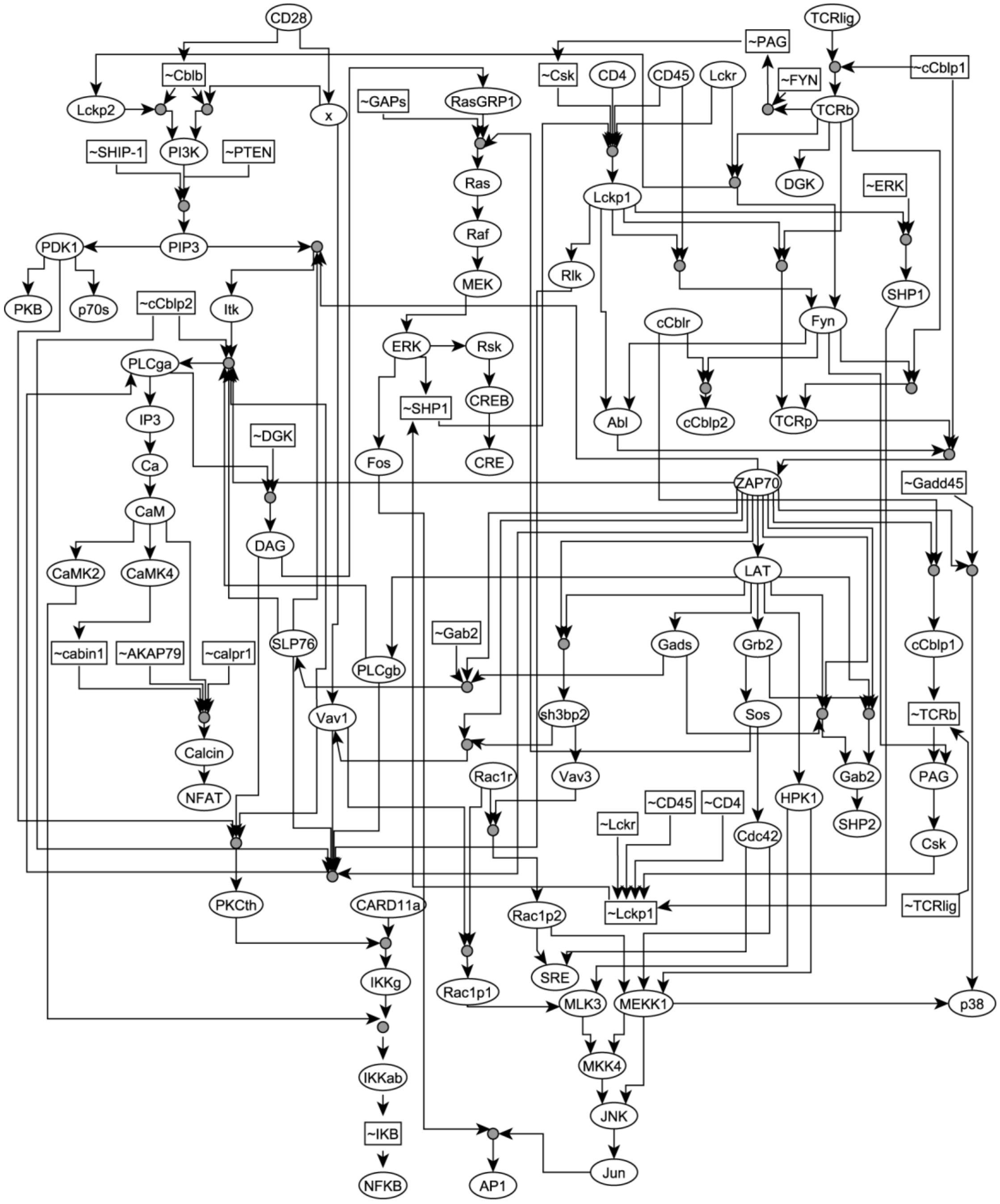

Figure S3. The expanded T cell receptor signaling network. Composite nodes are represented by small gray solid circles, original nodes are represented by large empty circles, and complementary nodes are represented by rectangles. The labels of complementary nodes are denoted by the labels for the corresponding original nodes with a symbol '~'.

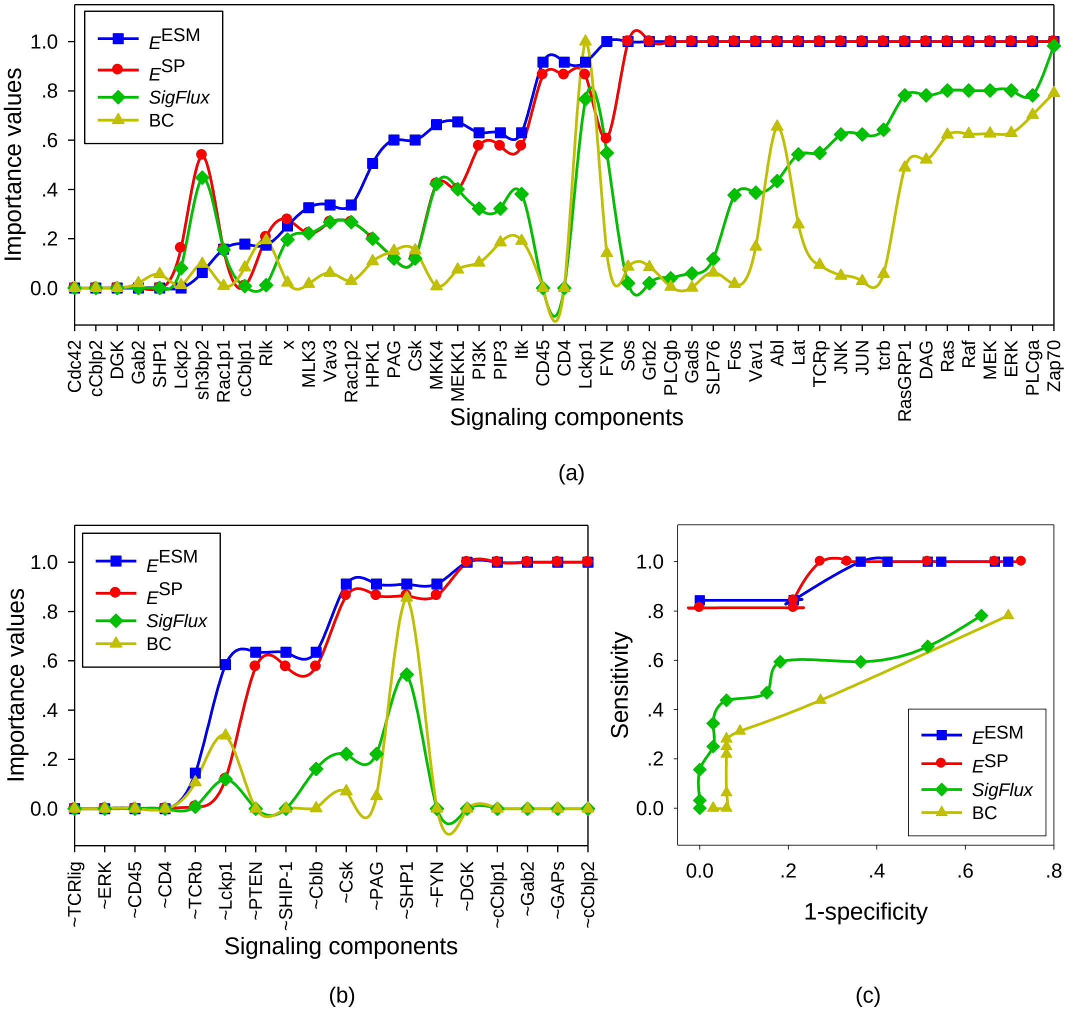

Figure S4. Comparison of different methods applied the T cell receptor signaling network with AP1 as the output. (a) Importance values obtained by single-node deletion of original nodes. (b) Importance values obtained by single-node deletion of complementary nodes. (c) Prediction accuracy. Rectangles indicate the importance values or prediction accuracy obtained by the ESM measure, circles represent the simple path measure, diamonds denote the *SigFlux* measure, and triangles show the betweenness centrality measure.
